# Environmental Risk Factors for Talaromycosis Hospitalizations of HIV-Infected Patients in Guangzhou, China: Case Crossover Study

**DOI:** 10.3389/fmed.2021.731188

**Published:** 2021-11-22

**Authors:** Yaping Wang, Kai Deng

**Affiliations:** Infectious Diseases Institute, Guangzhou Eighth People's Hospital, Guangzhou Medical University, Guangzhou, China

**Keywords:** talaromycosis, temperature, humidity, SO_2_, HIV

## Abstract

Talaromycosis is a fatal opportunistic infection prevalent in human immunodeficiency virus (HIV)-infected patients, previous studies suggest environmental humidity is associated with monthly talaromycosis hospitalizations of HIV-infected patients, but the acute risk factor remains uncertain. In this study, we evaluated the associations between talaromycosis hospitalizations of HIV-infected patients (*n* = 919) and environmental factors including meteorological variables and air pollutants at the event day (assumed “lag 0” since the exact infection date is hard to ascertain) and 1–7 days prior to event day (lag 1–lag 7) in conditional logistics regression models based on a case crossover design. We found that an interquartile range (IQR) increase in temperature at lag 0–lag 7 (odds ratio [OR] [95% CI] ranged from 1.748 [1.345–2.273] to 2.184 [1.672–2.854]), and an IQR increase in humidity at lag 0 (OR [95% CI] = 1.192 [1.052–1.350]), and lag 1 (OR [95% CI] = 1.199 [1.056–1.361]) were significantly associated with talaromycosis hospitalizations of HIV-infected patients. Besides, temperature was also a common predictor for talaromycosis in patients with co-infections including candidiasis (*n* = 386), *Pneumocystis* pneumonia (*n* = 183), pulmonary tuberculosis (*n* = 141), and chronic hepatitis (*n* = 158), while humidity was a specific risk factor for talaromycosis in patients with candidiasis, and an air pollutant, SO_2_, was a specific risk factor for talaromycosis in patients with *Pneumocystis* pneumonia. In an age stratified evaluation (cutoff = 50 years old), temperature was the only variable positively associated with talaromycosis in both younger and older patients. These findings broaden our understanding of the epidemiology and pathogenesis of talaromycosis in HIV-infected patients.

## Introduction

Talaromycosis is one of the most prevalent opportunistic infections among human immunodeficiency virus (HIV)-infected patients in South and Southeast Asia (1–3), and talaromycosis cases outside endemic areas were also reported partially due to increasing migration and travel to endemic regions, and the causes for some cases in non-endemic regions remain enigmatic (4–6). The prevalence of talaromycosis is closely associated with HIV infection (2, 7), e.g., in mainland China, 87.72% of talaromycosis occurs in HIV-infected patients (3). HIV-infected patients with a CD4 count lower than 100 cells per μL are susceptible to *Talaromyces marneffei*, the pathogen of talaromycosis, and have a high risk of death (8–10). The common clinical manifestations of talaromycosis include fever, weight loss, anemia, lymphadenopathy, and skin lesions (3, 10), and concomitant infections are frequent, particularly tuberculosis and salmonella infection (11, 12). The in-hospital mortality of HIV patients with talaromycosis was 17.5% in southern China, significantly higher than those without talaromycosis (7.6%) (9).

Although *Talaromyces marneffei* was first isolated from a bamboo rat early in 1956 and its mycology was described almost at the same time (2), knowledge regarding critical issues in talaromycosis epidemiology is still lacking. To date, there is no evidence of animal-to-human or human-to-human spread of talaromycosis. Thus, how a human host becomes infected remains elusive, and it was proposed that exposure to an unidentified common environmental reservoir may represent the transmission route (13, 14). Occupational exposure to soil, rainy season were considered as risk factors for talaromycosis, while consumption of bamboo rat was not (15). It was reported that a meteorological factor, humidity, was the most important predictor for monthly talaromycosis hospitalizations of HIV-infected patients in Vietnam based on Poisson regression analyses (16). However, whether meteorological factors have an acute effect on talaromycosis events remains uncertain. Besides, air pollutants have attracted more and more attention due to their significant associations with various infectious diseases including *Pneumocystis* pneumonia, an opportunistic infection in HIV patients (17, 18). Unfortunately, to our knowledge, by now there is no study regarding the associations between air pollutants and talaromycosis in HIV-infected patients.

Guangzhou is located in southern China with a humid subtropical climate. Guangzhou Eighth People's Hospital is the largest infectious disease hospital in Guangzhou and the training base for clinicians in HIV and acquired immune deficiency syndrome (AIDS). More than 80% of HIV-infected patients in Guangzhou were treated here during the last decade according to Guangzhou Centers for Disease Control and Prevention (CDC) reports (19). The main goal of the present study was to determine the acute associations between environmental factors including meteorological variables and air pollutants, and talaromycosis hospitalizations of HIV-infected patients based on a case crossover design.

## Materials and Methods

### Study Population

This study followed the guideline of the Ethics Committee of Guangzhou Eighth People's Hospital. The individual identifiers were not used in our study, so informed consent was not specifically required. We extracted information on the hospitalization date, diagnosis, CD4 cell count, age, sex from the records from January 1, 2014, through December 31, 2019. The patients who resided outside Guangzhou were excluded. Talaromycosis diagnosis was identified based on the International Classification of Diseases, 10th revision (ICD-10) codes B48.4, and confirmed by isolation of *Talaromyces marneffei* from bone marrow with standard culture techniques (16). In case of repeated hospitalizations, only the first event was included for analyses. Since the pathogens of co-infections among HIV-infected patients are usually part of the human saprophytic microflora, the diagnosis of co-infection was based on medical history, symptoms, physical examinations, and laboratory tests. To confirm *Cryptococcus neoformans* co-infection, body fluid (blood, cerebrospinal fluid, or sputum) was collected for culture, and a chest X-ray or computerized tomography scan of lungs, brain, or other parts of the body was conducted if necessary; for the diagnosis of candidiasis, samples from the infected body site (oral, skin, or vaginal discharge), blood, or lung biopsy were used for culture; sputum or bronchoalveolar lavage was collected for diagnosing *Pneumocystis* pneumonia; the diagnosis of pulmonary tuberculosis was established by isolation of mycobacterium tuberculosis from sputum or bronchoalveolar lavage.

### Meteorological and Air Pollution Data

Daily meteorological data, including temperature, humidity, wind speed, and pressure, were collected from Weather Underground, IBM (https://www.wunderground.com) from January 1, 2014, through December 31, 2019. Historical daily air pollution data, including coarse particulate matter (PM_10_), sulfur dioxide (SO_2_), carbon monoxide (CO), nitrogen dioxide (NO_2_), and ozone (O_3_) were obtained from the National Air Quality Study Platform (https://www.aqistudy.cn).

### Statistics Analysis

To analyze talaromycosis events in HIV patients, we employed a case crossover design. In this approach, the day of hospitalization date (event day, lag 0), and 1–7 days prior to event day (lag 1–lag7) were examined separately (20, 21). For each lag day, the control days were usually defined as the days on the same day of the week in the same month and year. This control strategy aiming to adjust for the effects of time-dependent confounding variables has been widely applied to evaluate acute risk factors for health outcomes (18, 22–24). Given that the incubation period of talaromycosis is 0–3 weeks (16), we adopted 21 and 28 days prior to event day as control days in this study.

To explore the environmental variables potentially associated with talaromycosis hospitalizations, we conducted a conditional logistics regression analysis. Odds ratios (OR) and 95% confidence interval (CI) were estimated for an interquartile range (IQR) increase in each environmental variable. The variables with *P* < 0.05 in univariate analysis were combined for multivariate analysis using the forward stepwise (likelihood ratio) method, and the variables with *P* < 0.05 were considered significant. All the statistical analyses were performed using IBM SPSS Statistics 26 (USA). Graphic representations were performed with GraphPad Prism 8 software.

## Results

### Study Subjects and Environmental Data

From January 2014 to December 2019, 919 consecutive HIV patients were admitted to Guangzhou Eighth People's Hospital with Talaromycosis (Table 1). All the patients had received antiviral therapy before admission. The median age of hospitalized patients was 39.6 years, and 81.7% (*n* = 751) were male. The median CD4 cell count was 9 cells/μL on admission. The most frequent co-infection was candidiasis (42.0%, including 381 oral candidiasis, 3 *Candida* pneumonia, 1 cutaneous candidiasis, and 1 vaginal candidiasis), followed by *Pneumocystis* pneumonia (19.9%), chronic hepatitis B or C (17.2%), pulmonary tuberculosis (15.3%), and *Cryptococcus neoformans* infection (3.2%). The main comorbidities included liver cirrhosis (2.4%) and diabetes (2.3%).

**Table 1:** Demographic and clinical features of 919 HIV-infected patients with talaromycosis.

**Demographic characteristic**	**Value**
Male	751 (81.7%)
Age, years	39.6 (30–48)
CD4 cell count, cells/μL	9 (4–21)
**Co-infection**	
*C. neoformans* infection	29 (3.2%)
Candidiasis	386 (42.0%)
*Pneumocystis* pneumonia	183 (19.9%)
Pulmonary tuberculosis	141 (15.3%)
Chronic hepatitis B or C	158 (17.2%)
**Comorbidity**	
Diabetes	21 (2.3%)
Liver cirrhosis	22 (2.4%)

*Data are in absolute count (%) for categorical variables and median (interquartile range [IQR]) for continuous data*.

The summary statistics for environmental variables from 2014 to 2019 were reported in Supplementary Table S1. There was a positive correlation between air pollutants (PM_10_, SO_2_, CO, NO_2_, and O_3_) except that CO was negatively correlated to O_3_ (*r* = −0.17, Supplementary Figure S1). Temperature was positively associated with O_3_ (*r* = 0.50) and negatively associated with other air pollutants. Humidity was positively and weakly correlated to CO and temperature (*r* = 0.09 and 0.16, respectively) and negatively correlated to other variables.

### Effects of Short Term Exposure to Environmental Variables on Talaromycosis Hospitalizations

**Figure 1:**
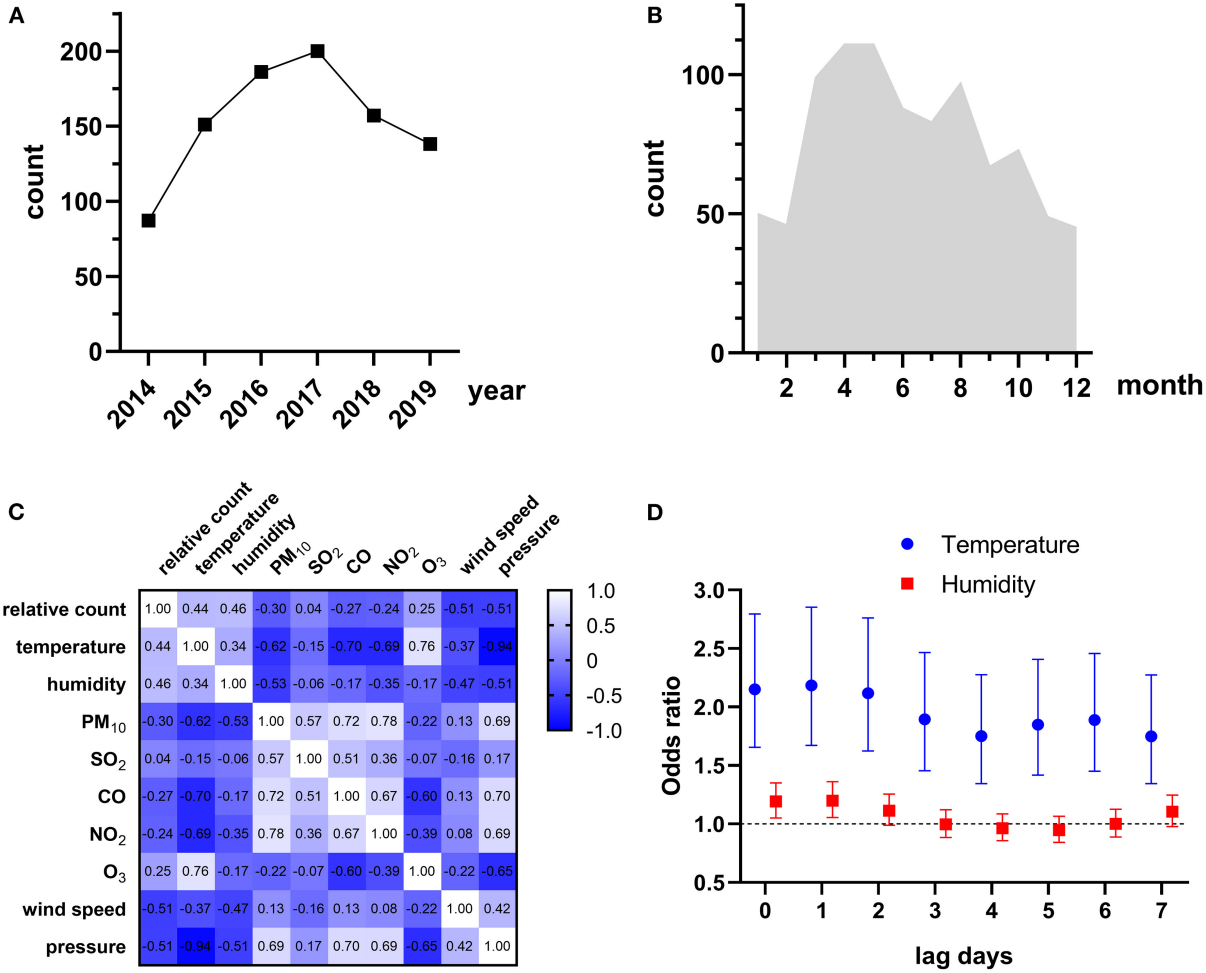
**(A)** Yearly talaromycosis hospitalizations of HIV-infected patients during 2014–2019. **(B)** Average monthly talaromycosis hospitalizations of HIV-infected patients. **(C)** Spearman's correlations between relative count of monthly talaromycosis hospitalizations and environmental variables. The correlation coefficient is shown in each cell. **(D)** Environmental variables significantly associated with talaromycosis hospitalizations.

The HIV admissions in Guangzhou Eighth People's Hospital increased rapidly from 1,395 to 2,767 during the study period, while the trend of talaromycosis hospitalizations of HIV-infected patients was not always consistent, which increased from 87 in 2014 to a peak (*n* = 200) in 2017, and gradually decreased to 138 in 2019 (Figure 1A). There were more talaromycosis hospitalizations during the warm season (average monthly hospitalizations = 91, from March to October) than the cold season (average monthly hospitalizations = 48, from November to February, Figure 1B). We defined the percentage of monthly hospitalizations counts in the same year as relative count, and found it was moderately and positively correlated to temperature (*r* = 0.44) and humidity (*r* = 0.46, Figure 1C and Supplementary Figure S2), and weakly correlated to O_3_ (*r* = 0.25).

To explore the environmental variables that associated with talaromycosis hospitalizations, we conducted a univariate analysis first and only the variables significant in univariate model (*P* < 0.05) were combined for multivariate analyses. The results were reported in Supplementary Table S2, and the environmental variables significantly associated with talaromycosis hospitalizations were shown in Figure 1D, that an IQR increase in temperature at lag 0 (OR [95% CI] = 2.151 [1.656–2.794]), lag 1 (OR [95% CI] = 2.184 [1.672–2.854]), lag 2 (OR [95% CI] = 2.117 [1.623–2.761]), lag 3 (OR [95% CI] = 1.895 [1.456–2.466]), lag 4 (OR [95% CI] = 1.750 [1.345–2.277]), lag 5 (OR [95% CI] = 1.848 [1.418–2.408]), lag 6 (OR [95% CI] = 1.888 [1.450–2.458]), and lag 7 (OR [95% CI] = 1.748 [1.345–2.273]), and an IQR increase in humidity at lag 0 (OR [95% CI] = 1.192 [1.052–1.350]) and lag1 (OR [95% CI] = 1.199 [1.056–1.361]) were significantly associated with talaromycosis hospitalizations of HIV-infected patients, while an IQR increase in humidity from lag 2 to lag 7 were not associated with talaromycosis event. These data suggested that temperature and humidity represent the acute risk factors for talaromycosis in HIV-infected patients.

### Risk Factors for Talaromycosis Hospitalizations of HIV-Infected Patients With Co-infections

**Figure 2:**
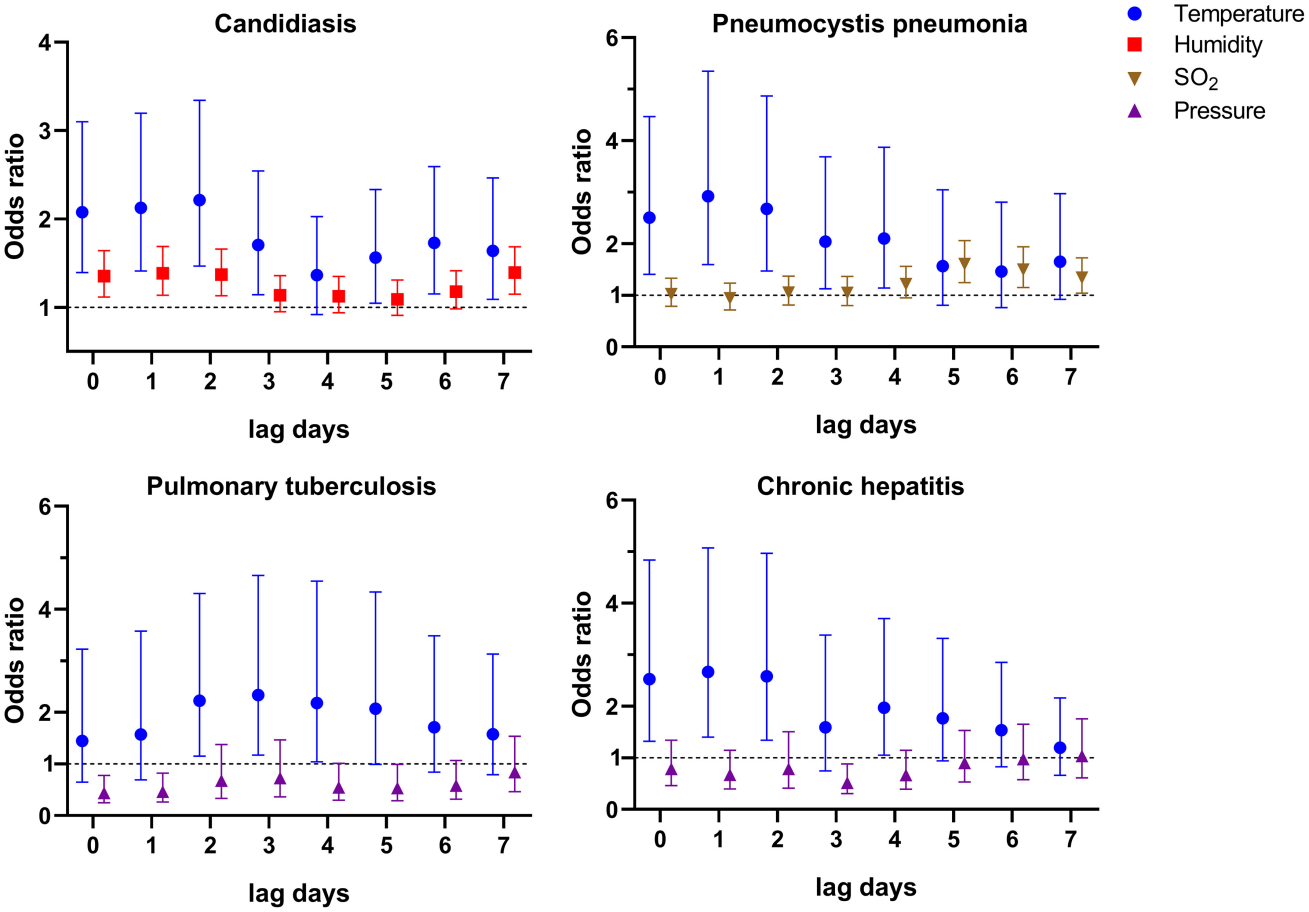
Environmental variables significantly associated with talaromycosis hospitalizations of HIV-infected patients with co-infections.

One of the most striking clinical features of HIV-infected patients is various co-infections including opportunistic infections, which are the leading cause of AIDS-related death (25, 26). In our cohort, there were approximately half of patients with candidiasis (42.0%), and nearly 1/5 of patients with *Pneumocystis* pneumonia (19.9%), chronic hepatitis B or C (17.2%), and pulmonary tuberculosis (15.3%). Thus, we also evaluated the risk factors for talaromycosis in patients with the four main co-infections (Supplementary Tables S3–S6), and the variables significantly associated with talaromycosis were shown in Figure 2. For patients with candidiasis, an IQR increase in temperature at lag 0–lag 3, and lag 5–lag 7 (OR [95% CI] ranged from 1.564 [1.048–2.332] to 2.214 [1.467–3.341]), and an IQR increase in humidity at lag 0–lag 2, and lag 7 (OR [95% CI] ranged from 1.354 [1.117–1.642] to 1.393 [1.150–1.687]) were significantly associated with talaromycosis; for patients with *Pneumocystis* pneumonia, an IQR increase in temperature at lag 0–lag 4 (OR [95% CI] ranged from 2.040 [1.128–3.689] to 2.924 [1.598–5.348]), and an IQR increase in an air pollutant, SO_2_, at lag 5 (OR [95% CI] = 1.605 [1.249–2.064]), lag 6 (OR [95% CI] = 1.495 [1.151–1.943]), and lag 7 (OR [95% CI] = 1.342 [1.043–1.726]) were significantly associated with talaromycosis; for patients with pulmonary tuberculosis, an IQR increase in temperature at lag 2-lag 4 (OR [95% CI] ranged from 2.180 [1.045–4.546] to 2.339 [1.174–4.659]) were positively associated with talaromycosis, while an IQR increase in pressure at lag 0–lag 1, and lag 5 (OR [95% CI] ranged from 0.439 [0.248–0.779] to 0.534 [0.287–0.991]) were negatively associated with talaromycosis; for patients with chronic hepatitis, an IQR increase in temperature at lag 0–lag 2, and lag 4 (OR [95% CI] ranged from 1.973 [1.051–3.704] to 2.666 [1.401–5.074]) were positively associated with talaromycosis, while an IQR increase in pressure at lag 3 (OR [95% CI] = 0.519 [0.305–0.882]) was negatively associated with talaromycosis. These results revealed temperature as a common predictor, as well as distinct risk factors for talaromycosis in HIV-infected patients with various co-infections.

### Risk Factors for Talaromycosis Hospitalizations of HIV-Infected Patients Stratified by Age

**Figure 3:**
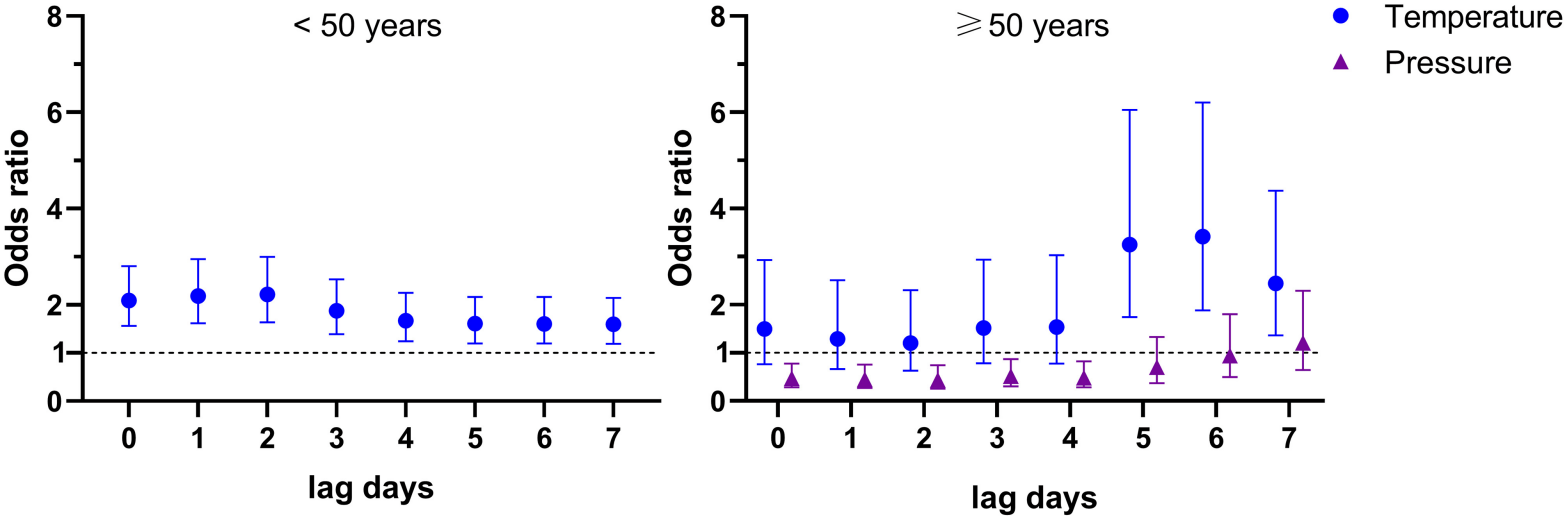
Environmental variables significantly associated with talaromycosis in younger (< 50 years) and older patients (≥ 50 years).

There are more and more elderly HIV-infected patients due to the improvements of antiretroviral therapy, e.g., the HIV-infected patients older than 50 years in the United States had increased to 51% in 2018 (27). However, the specific risk factors for talaromycosis in younger and older patients remain uncertain, thus, we here conducted an age stratified evaluation. In our cohort, there were 190 patients aged 50 and above (defined as “older patients”), and 729 patients younger than 50 years (defined as “younger patients”). The demographic and clinical data showed that there was no significant difference in gender distribution, CD4 cell count, and the frequency of co-infections and liver cirrhosis between younger and older patients, and more diabetes (younger vs. older: 1.5 vs. 5.3%, *P* = 0.005) in the older patients (Supplementary Table S7). Multivariate logistics regression results showed an IQR increase in temperature at lag 0–lag 7 (OR [95% CI] ranged from 1.598 [1.190–2.146] to 2.214 [1.636–2.994]) were significantly associated with talaromycosis in younger patients (Supplementary Table S8 and Figure 3); for older patients, an IQR increase in temperature at lag 5–lag 7 (OR [95% CI] ranged from 2.441 [1.364–4.368] to 3.419 [1.885–6.201]) were positively associated with talaromycosis, while an IQR increase in pressure at lag 0–lag 4 (OR [95% CI] ranged from 0.439 [0.259–0.745] to 0.515 [0.304–0.872]) were negatively associated with talaromycosis (Supplementary Table S9 and Figure 3). These data suggested that temperature was the common risk factor for talaromycosis in both younger and older patients, and pressure was a negative predictor for talaromycosis in older patients.

## Discussion

Previous studies have suggested meteorological variables like rainy season (12) and humidity (16) as predictors for monthly talaromycosis hospitalizations of HIV-infected patients. However, to our knowledge, the acute risk factors for talaromycosis are still elusive and warrant further investigation. Here, based on a case crossover design, we showed an IQR increase in humidity at lag 0 and lag 1 were significantly associated with talaromycosis hospitalizaitons of HIV-infected patients in Guangzhou, China (Figure 1D and Supplementary Table S2), consistent with previous findings. It was proposed that humidity may facilitate inhalation of infectious spores or hyphal fragment from the environmental reservoir driving talaromycosis pathogenesis (2, 16), our study adds new evidence of the theory. Besides, we also found an IQR increase in another meteorological variable, temperature, at lag 0–lag 7 were significantly associated with talaromycosis (Figure 1D). We noted that in the previous study conducted in Ho Chi Minh, Vietnam, temperature was not associated with monthly talaromycosis hospitalizations (16). This discrepancy may be explained by the distinct acute and subacute risk factors for talaromycosis, and different geographic regions. Interestingly, temperature was also considered as one of the most important variables in a maxent model recently established by Wei et al. (28) for predicting the distribution of global talaromycosis, supporting the results of our study. The median of CD4 counts in our cohort, which are closely related to talaromycosis pathogenesis (8–10), was as low as 9 cells/μL. Therefore, our cohort represented a population susceptible to talaromycosis.

We also showed that temperature was a common acute risk factor for talaromycosis in HIV-infected patients with four main co-infections, candidiasis, *Pneumocystis* pneumonia, pulmonary tuberculosis, and chronic hepatitis (Figure 2). By contrast, humidity was only associated with talaromycosis in patients with candidiasis but not other co-infections. It was reported that a humid environment favors the growth of *Candida* species, the pathogen of candidiasis, and promotes candidiasis pathogenesis (29). Thus, it seems that humidity is a common risk factor for talaromycosis and candidiasis. Another possible explanation is that a humid environment promotes candidiasis, and the latter is associated with talaromycosis progress, as we found humidity was not associated with talaromycosis in patients with the other three co-infections (Figure 2). Approximately half of the patients (42.0%) in our cohort were with candidiasis (Table 1), which may explain why humidity remained a risk factor for talaromycosis hospitalizations of all subjects (Figure 1D). In the previous studies suggesting humidity as a predictor for talaromycosis, 27.1% (139/513) of the patients were with oral candidiasis, but the proportion of other types of candidiasis was not mentioned (12, 16). Hence, this hypothesis warrants further studies. Due to a small number of patients with comorbidities, diabetes (*n* = 21) and liver cirrhosis (*n* = 22), we did not evaluate the risk factors for these two populations.

By now, whether increased levels of air pollutants, e.g., NO_2_, PM_10_, and SO_2_, the well-known risk factors for various infectious diseases (30–33), also affect the incidence of talaromycosis remains unclear. In this study, we showed that an IQR increase in SO_2_ at lag 5–lag 7 was significantly associated with talaromycosis hospitalizations of patients with *Pneumocystis* pneumonia. Thus, for the first time, we revealed a link between air pollutants and talaromycosis pathogenesis. It should be noted that SO_2_ also represents a risk factor for *Pneumocystis* pneumonia hospitalizations of HIV-infected patients (17), and by now how *Pneumocystis* pneumonia would affect the incidence of talaromycosis is not fully understood. Given that these two co-infections are prevalent in HIV-infected patients, the pathogenesis of talaromycosis is likely more complex than previously thought and further investigations are required to explore the relationship between SO_2_, talaromycosis, and *Pneumocystis* pneumonia. It is also worth noting that SO_2_ is more likely a driven factor but not the cause for talaromycosis hospitalizations. Exposure to SO_2_ leads to irritation of airway, bronchoconstriction, and dyspnea (34), which may facilitate talaromycosis pathogenesis, and the mechanism requires further research.

In the age stratified study, we found temperature was the only variable positively associated with talaromycosis in both younger and older HIV-infected patients (Figure 3). Since the gender distribution, CD4 cell count, and frequency of co-infections and liver cirrhosis were similar in the two groups (Supplementary Table S7), these data suggested that the age variable does not significantly affect the kinds of risk factors for talaromycosis. Interestingly, we found pressure was negatively associated with talaromycosis hospitalizations of the older patients (Figure 3), and patients with pulmonary tuberculosis or chronic hepatitis co-infection (Figure 2), but the underlying mechanism was not clear and warrants further research.

In the present study, we employed a case-crossover design, which has been widely used for evaluating predictors influencing the short-term health results, particularly acute infectious diseases (18, 20, 30, 35–37), as an alternative to Poisson distribution model to explore the associations between environmental variables and talaromycosis hospitalizations, and successfully identified the specific meteorological variables and air pollutant associated with talaromycosis in HIV-infected patients (Figures 1D, 2), suggesting the feasibility of this method. Unlike previous studies using the counts of talaromycosis hospitalizations aggregated by week or by month (16), we here showed that acute exposure to the risk factors at a single day level (lag 0–lag 7) was significantly associated with talaromycosis hospitalizations of HIV-infected patients, which would broaden our understanding of the epidemiology and pathogenesis of the opportunistic infection.

Our study is limited in that it was a single-center study and some patients with talaromycosis treated elsewhere were not included. However, since more than 80% of HIV-infected patients in Guangzhou were treated in Guangzhou Eighth People's Hospital according to Guangzhou CDC report (19), and a large sample size included in this study enabled overcoming this drawback and accurate assessment of the risk factors for talaromycosis.

In summary, our study confirmed an acute association between environmental variables and talaromycosis onset and also revealed the air pollutant as a specific risk factor for talaromycosis in a subpopulation with co-infection, which would undoubtedly provide clues for a greater understanding of the transmission and pathogenesis of the opportunistic infection prevalent in HIV-infected patients.

## Data Availability Statement

The raw data supporting the conclusions of this article will be made available by the authors, without undue reservation.

## Ethics Statement

The studies involving human participants were reviewed and approved by Ethics Committee of Guangzhou Eighth People's Hospital. Written informed consent for participation was not required for this study in accordance with the national legislation and the institutional requirements.

## Author Contributions

KD conceived the study and supervised all aspects of the study. YW and KD collected and analyzed the data and prepared the manuscript. All authors contributed to the article and approved the submitted version.

## Funding

This study was supported by the Natural Science Foundation of Guangdong Province (2017A030310035).

## Conflict of Interest

The authors declare that the research was conducted in the absence of any commercial or financial relationships that could be construed as a potential conflict of interest.

## Publisher's Note

All claims expressed in this article are solely those of the authors and do not necessarily represent those of their affiliated organizations, or those of the publisher, the editors and the reviewers. Any product that may be evaluated in this article, or claim that may be made by its manufacturer, is not guaranteed or endorsed by the publisher.

## Supplementary Material

Supplementary Figure S1 Correlations between environmental variables. The Pearson correlation coefficient is shown in each cell.

Supplementary Figure S2 Dynamics of relative count, humidity, and temperature during 2014–2019.

Supplementary Table S1 Summary statistics for environmental variables in Guangzhou, 2014–2019.

Supplementary Table S2 Associations between talaromycosis hospitalizations and an IQR increase in environmental variables.

Supplementary Table S3 Associations between talaromycosis hospitalizations of HIV-infected patients with candidiasis and an IQR increase in environmental variables.

Supplementary Table S4 Associations between talaromycosis hospitalizations of HIV-infected patients with *Pneumocystis* pneumonia and an IQR increase in environmental variables.

Supplementary Table S5 Associations between talaromycosis hospitalizations of HIV-infected patients with pulmonary tuberculosis and an IQR increase in environmental variables.

Supplementary Table S6 Associations between talaromycosis hospitalizations of HIV-infected patients with chronic hepatitis B or C and an IQR increase in environmental variables.

Supplementary Table S7 Demographic and clinical features of patients stratified by age.

Supplementary Table S8 Associations between talaromycosis hospitalizations of younger patients and an IQR increase in environmental variables.

Supplementary Table S9 Associations between talaromycosis hospitalizations of older patients and an IQR increase in environmental variables.
