# Supplementary figures and images for: Environmental Risk Factors for Talaromycosis Hospitalizations of HIV-Infected Patients in Guangzhou, China: Case Crossover Study
Source: Front Med (Lausanne). 2021 Nov 22;8:731188. doi: 10.3389/fmed.2021.731188 (PMC8645774; doi:10.3389/fmed.2021.731188)

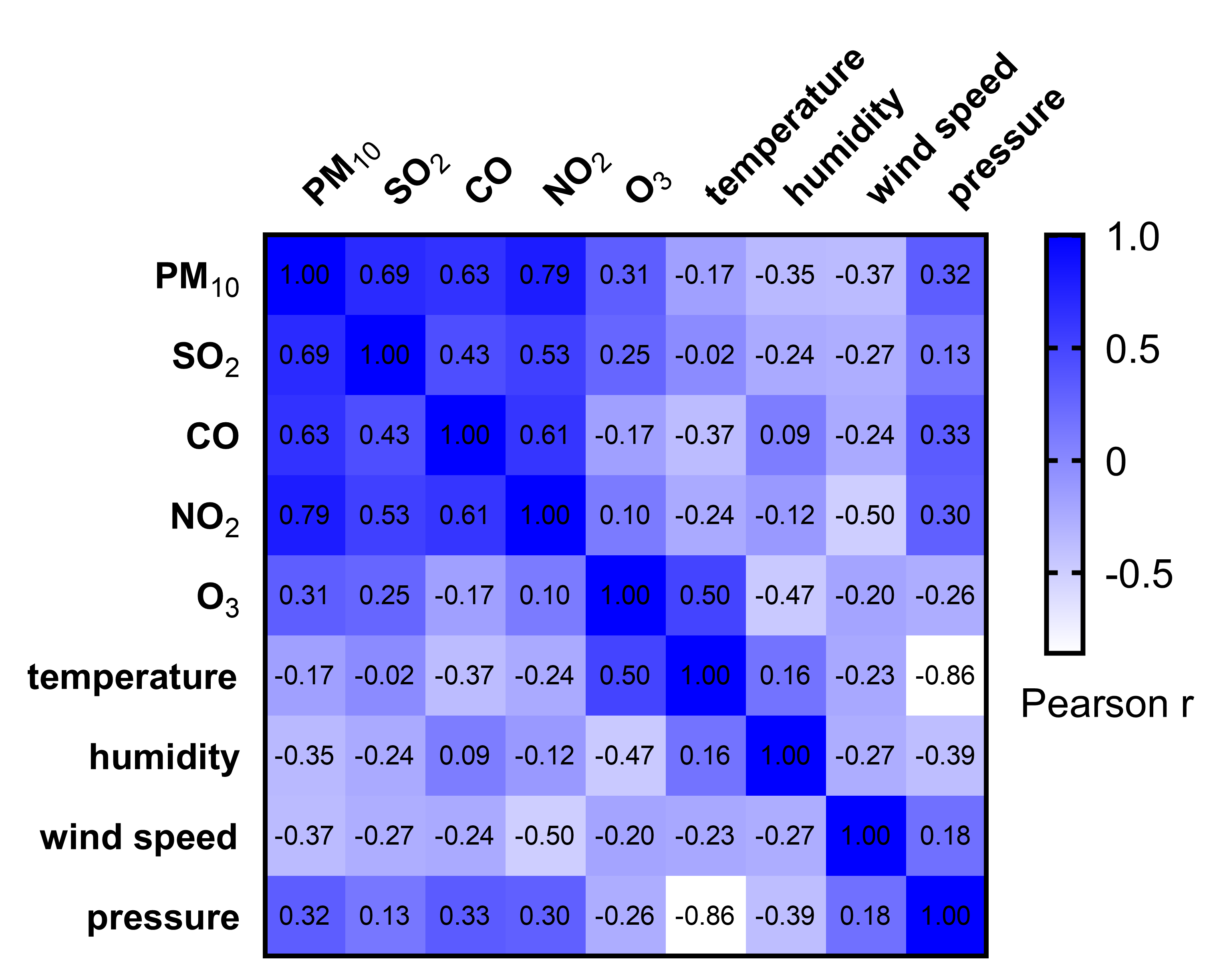

Supplement: Supplementary Figure S1 — Correlations between environmental variables. The Pearson correlation coefficient is shown in each cell. [file Image_1.TIF]

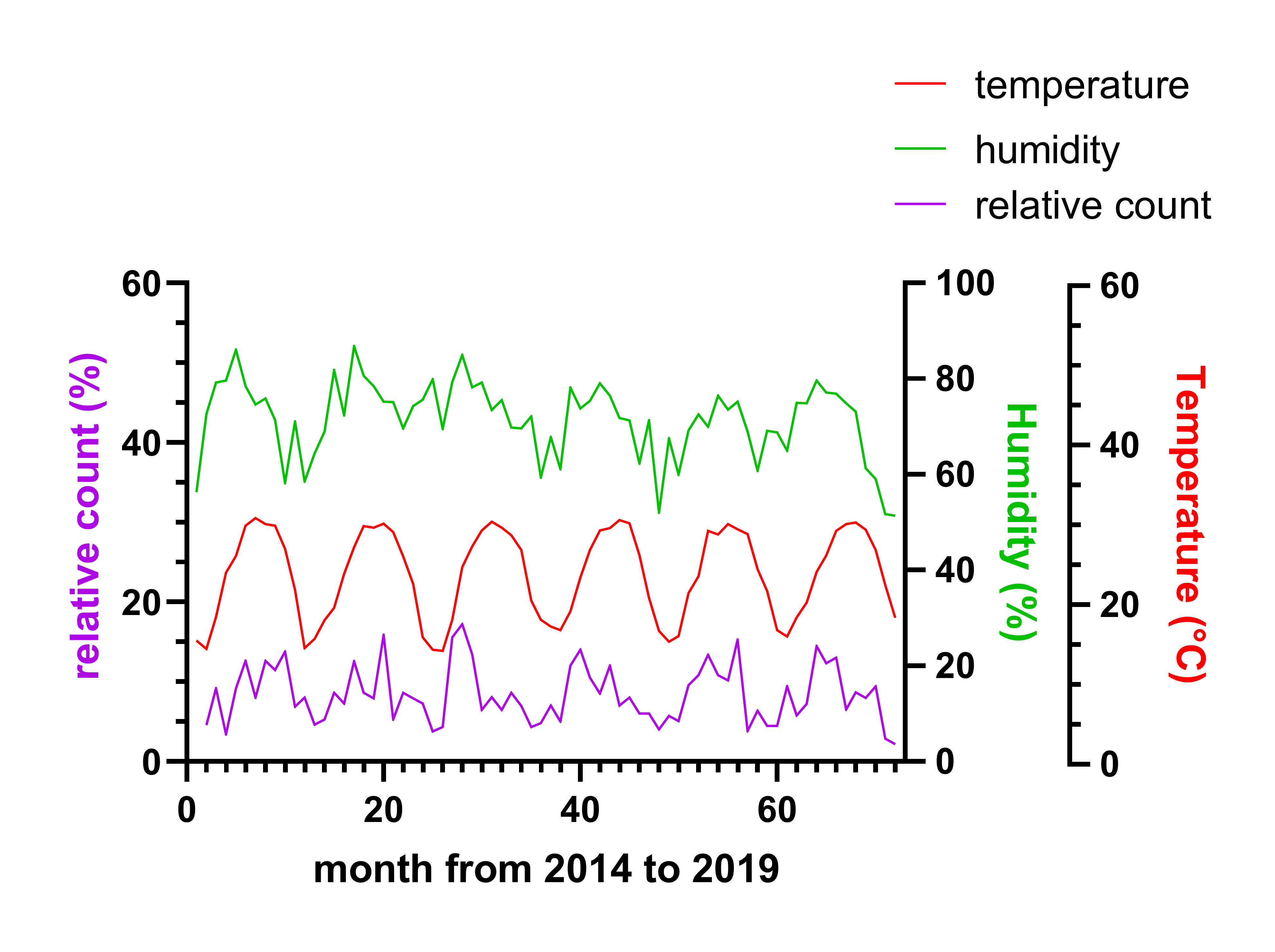

Supplement: Supplementary Figure S2 — Dynamics of relative count, humidity, and temperature during 2014–2019. [file Image_2.TIF]
