# Supplementary material for: Environmental Risk Factors for Talaromycosis Hospitalizations of HIV-Infected Patients in Guangzhou, China: Case Crossover Study
Source: Front Med (Lausanne). 2021 Nov 22;8:731188. doi: 10.3389/fmed.2021.731188 (PMC8645774; doi:10.3389/fmed.2021.731188)
Supplement: Supplementary Table S1 — Summary statistics for environmental variables in Guangzhou, 2014–2019. [file Table_1.DOCX]

Table S1. Summary statistics of environmental variables in Guangzhou, 2014-2019.

|  | Minimum |  | Percentiles | | | Maximum | Mean ± SD | IQR |
| --- | --- | --- | --- | --- | --- | --- | --- | --- |
|  |  |  | 25% | 50% | 75% |  |  |  |
| PM_10_ (μg/m^3^) | 10 |  | 37 | 51 | 74 | 212 | 58.1 ± 28.4 | 37 |
| SO_2_ (μg/m^3^) | 3 |  | 8 | 11 | 14 | 42 | 11.6 ± 5.3 | 6 |
| CO (mg/m^3^) | 0.5 |  | 0.8 | 0.9 | 1 | 2.6 | 0.9 ± 0.2 | 0.2 |
| NO_2_ (μg/m^3^) | 8 |  | 34 | 43 | 56 | 178 | 47.2 ± 19.1 | 22 |
| O_3_ (μg/m^3^) | 0 |  | 50 | 86 | 126 | 287 | 91.8 ± 52.5 | 76 |
| Temperature (℃) | 4.6 |  | 18.9 | 25 | 28.7 | 34.2 | 23.6 ± 6.2 | 9.8 |
| Humidity (%) | 18.1 |  | 64 | 72.8 | 81.7 | 99.3 | 71.5 ± 13.7 | 17.7 |
| Wind speed (mph) | 0 |  | 6.7 | 8.5 | 11.6 | 39.6 | 9.6 ± 4.4 | 4.9 |
| Pressure (hPa) | 991.8 |  | 1005.3 | 1010.8 | 1016.2 | 1035.2 | 1010.9 ± 7.1 | 10.9 |

Abbreviations: SD, Standard deviation; IQR, interquartile range; PM_10_, coarse particulate matter; mph, mile per hour; hPa, hectopascal.
