# Supplementary material for: Environmental Risk Factors for Talaromycosis Hospitalizations of HIV-Infected Patients in Guangzhou, China: Case Crossover Study
Source: Front Med (Lausanne). 2021 Nov 22;8:731188. doi: 10.3389/fmed.2021.731188 (PMC8645774; doi:10.3389/fmed.2021.731188)
Supplement: Supplementary Table S2 — Associations between talaromycosis hospitalizations and an IQR increase in environmental variables. [file Table_2.DOCX]

Table S2. Associations between talaromycosis hospitalizations and an IQR increase in environmental variables.

| Variable | Univariate analysis | |  | Multivariate analysis | |
| --- | --- | --- | --- | --- | --- |
|  | OR (95% CI) | P value |  | OR (95% CI) | P value |
| lag 0 |  |  |  |  |  |
| PM_10_ (μg/m^3^) | 0.965 (0.856-1.088) | 0.566 |  | .. | .. |
| SO_2_ (μg/m^3^) | 0.954 (0.842-1.080) | 0.457 |  | .. | .. |
| CO (mg/m^3^) | 0.997 (0.914-1.087) | 0.941 |  | .. | .. |
| NO_2_ (μg/m^3^) | 0.995 (0.892-1.110) | 0.928 |  | .. | .. |
| O_3_ (μg/m^3^) | 0.954 (0.841-1.081) | 0.459 |  | .. | .. |
| Temperature (℃) | 2.096 (1.617-2.716) | <0.001 |  | 2.151 (1.656-2.794) | <0.001 |
| Humidity (%) | 1.159 (1.028-1.306) | 0.016 |  | 1.192 (1.052-1.350) | 0.006 |
| Wind speed (mph) | 0.965 (0.879-1.060) | 0.461 |  | .. | .. |
| Pressure (hPa) | 0.656 (0.523-0.822) | <0.001 |  | .. | .. |
| lag 1 |  |  |  |  |  |
| PM_10_ (μg/m^3^) | 0.991 (0.879-1.117) | 0.882 |  | .. | .. |
| SO_2_ (μg/m^3^) | 0.992 (0.877-1.122) | 0.903 |  | .. | .. |
| CO (mg/m^3^) | 1.002 (0.919-1.093) | 0.965 |  | .. | .. |
| NO_2_ (μg/m^3^) | 1.003 (0.900-1.118) | 0.959 |  | .. | .. |
| O_3_ (μg/m^3^) | 0.974 (0.860-1.104) | 0.681 |  | .. | .. |
| Temperature (℃) | 2.124 (1.630-2.767) | <0.001 |  | 2.184 (1.672-2.854) | <0.001 |
| Humidity (%) | 1.164 (1.031-1.315) | 0.014 |  | 1.199 (1.056-1.361) | 0.005 |
| Wind speed (mph) | 0.928 (0.842-1.022) | 0.127 |  | .. | .. |
| Pressure (hPa) | 0.642 (0.510-0.807) | <0.001 |  | .. | .. |
| lag 2 |  |  |  |  |  |
| PM_10_ (μg/m^3^) | 1.076 (0.956-1.210) | 0.225 |  | .. | .. |
| SO_2_ (μg/m^3^) | 1.098 (0.970-1.244) | 0.139 |  | .. | .. |
| CO (mg/m^3^) | 1.038 (0.953-1.131) | 0.390 |  | .. | .. |
| NO_2_ (μg/m^3^) | 1.061 (0.953-1.181) | 0.281 |  | .. | .. |
| O_3_ (μg/m^3^) | 1.003 (0.888-1.134) | 0.958 |  | .. | .. |
| Temperature (℃) | 2.117 (1.623-2.761) | <0.001 |  | 2.117 (1.623-2.761) | <0.001 |
| Humidity (%) | 1.114 (0.989-1.256) | 0.075 |  | .. | .. |
| Wind speed (mph) | 0.863 (0.783-0.952) | 0.003 |  | .. | .. |
| Pressure (hPa) | 0.622 (0.494-0.784) | <0.001 |  | .. | .. |
| lag 3 |  |  |  |  |  |
| PM_10_ (μg/m^3^) | 1.023 (0.906-1.156) | 0.714 |  | .. | .. |
| SO_2_ (μg/m^3^) | 1.028 (0.907-1.165) | 0.667 |  | .. | .. |
| CO (mg/m^3^) | 0.966 (0.885-1.055) | 0.447 |  | .. | .. |
| NO_2_ (μg/m^3^) | 0.979 (0.875-1.094) | 0.706 |  | .. | .. |
| O_3_ (μg/m^3^) | 1.033 (0.911-1.171) | 0.609 |  | .. | .. |
| Temperature (℃) | 1.895 (1.456-2.466) | <0.001 |  | 1.895 (1.456-2.466) | <0.001 |
| Humidity (%) | 0.996 (0.885-1.122) | 0.951 |  | .. | .. |
| Wind speed (mph) | 0.891 (0.808-0.981) | 0.019 |  | .. | .. |
| Pressure (hPa) | 0.676 (0.536-0.851) | 0.001 |  | .. | .. |
| lag 4 |  |  |  | .. | .. |
| PM_10_ (μg/m^3^) | 1.006 (0.890-1.137) | 0.920 |  | .. | .. |
| SO_2_ (μg/m^3^) | 1.055 (0.934-1.192) | 0.387 |  | .. | .. |
| CO (mg/m^3^) | 0.963 (0.883-1.051) | 0.401 |  | .. | .. |
| NO_2_ (μg/m^3^) | 0.941 (0.839-1.054) | 0.292 |  | .. | .. |
| O_3_ (μg/m^3^) | 1.046 (0.924-1.184) | 0.477 |  | .. | .. |
| Temperature (℃) | 1.750 (1.345-2.277) | <0.001 |  | 1.750 (1.345-2.277) | <0.001 |
| Humidity (%) | 0.964 (0.857-1.086) | 0.549 |  | .. | .. |
| Wind speed (mph) | 0.962 (0.874-1.059) | 0.433 |  | .. | .. |
| Pressure (hPa) | 0.714 (0.566-0.900) | 0.004 |  | .. | .. |
| lag 5 |  |  |  | .. | .. |
| PM_10_ (μg/m^3^) | 1.030 (0.913-1.161) | 0.633 |  | .. | .. |
| SO_2_ (μg/m^3^) | 1.109 (0.983-1.250) | 0.092 |  | .. | .. |
| CO (mg/m^3^) | 0.976 (0.896-1.064) | 0.589 |  | .. | .. |
| NO_2_ (μg/m^3^) | 0.972 (0.87-1.086) | 0.619 |  | .. | .. |
| O_3_ (μg/m^3^) | 1.027 (0.907-1.163) | 0.672 |  | .. | .. |
| Temperature (℃) | 1.848 (1.418-2.408) | <0.001 |  | 1.848 (1.418-2.408) | <0.001 |
| Humidity (%) | 0.947 (0.842-1.066) | 0.369 |  | .. | .. |
| Wind speed (mph) | 1.011 (0.920-1.110) | 0.821 |  | .. | .. |
| Pressure (hPa) | 0.734 (0.582-0.925) | 0.009 |  | .. | .. |
| lag 6 |  |  |  | .. | .. |
| PM_10_ (μg/m^3^) | 1.010 (0.896-1.137) | 0.875 |  | .. | .. |
| SO_2_ (μg/m^3^) | 1.024 (0.907-1.157) | 0.701 |  | .. | .. |
| CO (mg/m^3^) | 0.989 (0.908-1.077) | 0.805 |  | .. | .. |
| NO_2_ (μg/m^3^) | 0.988 (0.887-1.100) | 0.827 |  | .. | .. |
| O_3_ (μg/m^3^) | 0.977 (0.861-1.107) | 0.711 |  | .. | .. |
| Temperature (℃) | 1.888 (1.450-2.458) | <0.001 |  | 1.888 (1.450-2.458) | <0.001 |
| Humidity (%) | 1.001 (0.889-1.126) | 0.992 |  | .. | .. |
| Wind speed (mph) | 0.995 (0.905-1.093) | 0.910 |  | .. | .. |
| Pressure (hPa) | 0.735 (0.583-0.925) | 0.009 |  | .. | .. |
| lag 7 |  |  |  | .. | .. |
| PM_10_ (μg/m^3^) | 0.988 (0.878-1.111) | 0.836 |  | .. | .. |
| SO_2_ (μg/m^3^) | 0.990 (0.874-1.120) | 0.869 |  | .. | .. |
| CO (mg/m^3^) | 1.025 (0.941-1.118) | 0.567 |  | .. | .. |
| NO_2_ (μg/m^3^) | 0.983 (0.882-1.095) | 0.756 |  | .. | .. |
| O_3_ (μg/m^3^) | 0.934 (0.823-1.060) | 0.290 |  | .. | .. |
| Temperature (℃) | 1.748 (1.345-2.273) | <0.001 |  | 1.748 (1.345-2.273) | <0.001 |
| Humidity (%) | 1.105 (0.979-1.247) | 0.105 |  | .. | .. |
| Wind speed (mph) | 0.936 (0.852-1.029) | 0.174 |  | .. | .. |
| Pressure (hPa) | 0.752 (0.595-0.950) | 0.017 |  | .. | .. |

Abbreviations: IQR, interquartile range; PM_10_, coarse particulate matter; OR, odds ratio; CI, confidence interval; mph, mile per hour; hPa, hectopascal.
