# Supplementary material for: Environmental Risk Factors for Talaromycosis Hospitalizations of HIV-Infected Patients in Guangzhou, China: Case Crossover Study
Source: Front Med (Lausanne). 2021 Nov 22;8:731188. doi: 10.3389/fmed.2021.731188 (PMC8645774; doi:10.3389/fmed.2021.731188)
Supplement: Supplementary Table S3 — Associations between talaromycosis hospitalizations of HIV-infected patients with candidiasis and an IQR increase in environmental variables. [file Table_3.DOCX]

Table S3. Associations between talaromycosis hospitalizations of HIV-infected patients with candidiasis and an IQR increase in environmental variables.

| Variable | Univariate analysis | |  | Multivariate analysis | |
| --- | --- | --- | --- | --- | --- |
|  | OR (95% CI) | P value |  | OR (95% CI) | P value |
| lag 0 |  |  |  |  |  |
| PM_10_ (μg/m^3^) | 0.920 (0.767-1.105) | 0.373 |  | .. | .. |
| SO_2_ (μg/m^3^) | 0.936 (0.777-1.128) | 0.487 |  | .. | .. |
| CO (mg/m^3^) | 0.974 (0.848-1.120) | 0.714 |  | .. | .. |
| NO_2_ (μg/m^3^) | 0.976 (0.829-1.150) | 0.774 |  | .. | .. |
| O_3_ (μg/m^3^) | 0.946 (0.779-1.151) | 0.581 |  | .. | .. |
| Temperature (℃) | 1.972 (1.332-2.921) | 0.001 |  | 2.077 (1.393-3.099) | <0.001 |
| Humidity (%) | 1.310 (1.089-1.575) | 0.004 |  | 1.354 (1.117-1.642) | 0.002 |
| Wind speed (mph) | 0.992 (0.862-1.141) | 0.905 |  | .. | .. |
| Pressure (hPa) | 0.630 (0.446-0.891) | 0.009 |  | .. | .. |
| lag 1 |  |  |  |  |  |
| PM_10_ (μg/m^3^) | 0.917 (0.764-1.102) | 0.357 |  | .. | .. |
| SO_2_ (μg/m^3^) | 0.925 (0.765-1.118) | 0.422 |  | .. | .. |
| CO (mg/m^3^) | 0.954 (0.831-1.096) | 0.506 |  | .. | .. |
| NO_2_ (μg/m^3^) | 0.956 (0.812-1.125) | 0.588 |  | .. | .. |
| O_3_ (μg/m^3^) | 0.987 (0.812-1.199) | 0.894 |  | .. | .. |
| Temperature (℃) | 1.989 (1.334-2.965) | 0.001 |  | 2.125 (1.412-3.197) | <0.001 |
| Humidity (%) | 1.329 (1.101-1.605) | 0.003 |  | 1.386 (1.137-1.689) | 0.001 |
| Wind speed (mph) | 0.943 (0.814-1.093) | 0.437 |  | .. | .. |
| Pressure (hPa) | 0.642 (0.453-0.910) | 0.013 |  | .. | .. |
| lag 2 |  |  |  |  |  |
| PM_10_ (μg/m^3^) | 1.085 (0.910-1.292) | 0.363 |  | .. | .. |
| SO_2_ (μg/m^3^) | 1.116 (0.923-1.348) | 0.257 |  | .. | .. |
| CO (mg/m^3^) | 1.118 (0.979-1.275) | 0.099 |  | .. | .. |
| NO_2_ (μg/m^3^) | 1.032 (0.878-1.213) | 0.701 |  | .. | .. |
| O_3_ (μg/m^3^) | 1.002 (0.830-1.209) | 0.987 |  | .. | .. |
| Temperature (℃) | 2.036 (1.363-3.041) | 0.001 |  | 2.214 (1.467-3.341) | <0.001 |
| Humidity (%) | 1.302 (1.086-1.562) | 0.004 |  | 1.370 (1.132-1.659) | 0.001 |
| Wind speed (mph) | 0.843 (0.724-0.982) | 0.028 |  | .. | .. |
| Pressure (hPa) | 0.627 (0.442-0.889) | 0.009 |  | .. | .. |
| lag 3 |  |  |  |  |  |
| PM_10_ (μg/m^3^) | 1.074 (0.896-1.286) | 0.441 |  | .. | .. |
| SO_2_ (μg/m^3^) | 1.054 (0.868-1.280) | 0.597 |  | .. | .. |
| CO (mg/m^3^) | 1.078 (0.942-1.233) | 0.276 |  | .. | .. |
| NO_2_ (μg/m^3^) | 1.006 (0.852-1.188) | 0.941 |  | .. | .. |
| O_3_ (μg/m^3^) | 0.955 (0.786-1.161) | 0.644 |  | .. | .. |
| Temperature (℃) | 1.705 (1.143-2.544) | 0.009 |  | 1.705 (1.143-2.544) | 0.009 |
| Humidity (%) | 1.137 (0.951-1.358) | 0.159 |  | .. | .. |
| Wind speed (mph) | 0.825 (0.707-0.962) | 0.014 |  | .. | .. |
| Pressure (hPa) | 0.700 (0.492-0.995) | 0.047 |  | .. | .. |
| lag 4 |  |  |  |  |  |
| PM_10_ (μg/m^3^) | 0.973 (0.813-1.165) | 0.765 |  | .. | .. |
| SO_2_ (μg/m^3^) | 1.017 (0.846-1.223) | 0.859 |  | .. | .. |
| CO (mg/m^3^) | 1.038 (0.907-1.188) | 0.588 |  | .. | .. |
| NO_2_ (μg/m^3^) | 0.941 (0.795-1.114) | 0.479 |  | .. | .. |
| O_3_ (μg/m^3^) | 0.963 (0.793-1.169) | 0.704 |  | .. | .. |
| Temperature (℃) | 1.364 (0.918-2.028) | 0.125 |  | .. | .. |
| Humidity (%) | 1.126 (0.940-1.349) | 0.197 |  | .. | .. |
| Wind speed (mph) | 0.908 (0.782-1.054) | 0.204 |  | .. | .. |
| Pressure (hPa) | 0.816 (0.572-1.163) | 0.261 |  | .. | .. |
| lag 5 |  |  |  |  |  |
| PM_10_ (μg/m^3^) | 0.965 (0.806-1.155) | 0.697 |  | .. | .. |
| SO_2_ (μg/m^3^) | 1.024 (0.854-1.229) | 0.796 |  | .. | .. |
| CO (mg/m^3^) | 1.029 (0.897-1.181) | 0.681 |  | .. | .. |
| NO_2_ (μg/m^3^) | 0.977 (0.829-1.152) | 0.782 |  | .. | .. |
| O_3_ (μg/m^3^) | 0.941 (0.776-1.141) | 0.537 |  | .. | .. |
| Temperature (℃) | 1.564 (1.048-2.332) | 0.028 |  | 1.564 (1.048-2.332) | 0.028 |
| Humidity (%) | 1.092 (0.910-1.310) | 0.344 |  | .. | .. |
| Wind speed (mph) | 1.022 (0.885-1.181) | 0.764 |  | .. | .. |
| Pressure (hPa) | 0.802 (0.562-1.144) | 0.223 |  | .. | .. |
| lag 6 |  |  |  |  |  |
| PM_10_ (μg/m^3^) | 0.979 (0.820-1.170) | 0.818 |  | .. | .. |
| SO_2_ (μg/m^3^) | 0.983 (0.818-1.183) | 0.859 |  | .. | .. |
| CO (mg/m^3^) | 1.030 (0.899-1.180) | 0.668 |  | .. | .. |
| NO_2_ (μg/m^3^) | 1.000 (0.849-1.177) | 0.998 |  | .. | .. |
| O_3_ (μg/m^3^) | 0.934 (0.769-1.135) | 0.494 |  | .. | .. |
| Temperature (℃) | 1.729 (1.153-2.594) | 0.008 |  | 1.729 (1.153-2.594) | 0.008 |
| Humidity (%) | 1.178 (0.982-1.413) | 0.077 |  | .. | .. |
| Wind speed (mph) | 0.956 (0.825-1.107) | 0.547 |  | .. | .. |
| Pressure (hPa) | 0.714 (0.500-1.021) | 0.065 |  | .. | .. |
| lag 7 |  |  |  |  |  |
| PM_10_ (μg/m^3^) | 0.951 (0.798-1.135) | 0.578 |  |  |  |
| SO_2_ (μg/m^3^) | 0.910 (0.753-1.101) | 0.332 |  |  |  |
| CO (mg/m^3^) | 1.057 (0.921-1.213) | 0.432 |  |  |  |
| NO_2_ (μg/m^3^) | 1.023 (0.873-1.198) | 0.781 |  |  |  |
| O_3_ (μg/m^3^) | 0.844 (0.692-1.030) | 0.095 |  |  |  |
| Temperature (℃) | 1.535 (1.027-2.294) | 0.036 |  | 1.640 (1.092-2.465) | 0.017 |
| Humidity (%) | 1.357 (1.126-1.635) | 0.001 |  | 1.393 (1.150-1.687) | 0.001 |
| Wind speed (mph) | 0.872 (0.750-1.014) | 0.076 |  |  |  |
| Pressure (hPa) | 0.751 (0.523-1.078) | 0.121 |  |  |  |

Abbreviations: IQR, interquartile range; PM_10_, coarse particulate matter; OR, odds ratio; CI, confidence interval; mph, mile per hour; hPa, hectopascal.
