# Supplementary material for: Environmental Risk Factors for Talaromycosis Hospitalizations of HIV-Infected Patients in Guangzhou, China: Case Crossover Study
Source: Front Med (Lausanne). 2021 Nov 22;8:731188. doi: 10.3389/fmed.2021.731188 (PMC8645774; doi:10.3389/fmed.2021.731188)
Supplement: Supplementary Table S4 — Associations between talaromycosis hospitalizations of HIV-infected patients with Pneumocystis pneumonia and an IQR increase in environmental variables. [file Table_4.DOCX]

Table S4. Associations between talaromycosis hospitalizations of HIV-infected patients with *Pneumocystis* pneumonia and an IQR increase in environmental variables.

| Variable | Univariate analysis | |  | Multivariate analysis | |
| --- | --- | --- | --- | --- | --- |
|  | OR (95% CI) | P value |  | OR (95% CI) | P value |
| lag 0 |  |  |  |  |  |
| PM_10_ (μg/m^3^) | 0.969 (0.741-1.266) | 0.816 |  | .. | .. |
| SO_2_ (μg/m^3^) | 1.022 (0.786-1.330) | 0.870 |  | .. | .. |
| CO (mg/m^3^) | 0.889 (0.729-1.083) | 0.244 |  | .. | .. |
| NO_2_ (μg/m^3^) | 0.949 (0.737-1.223) | 0.687 |  | .. | .. |
| O_3_ (μg/m^3^) | 0.934 (0.699-1.247) | 0.643 |  | .. | .. |
| Temperature (℃) | 2.508 (1.408-4.468) | 0.002 |  | 2.508 (1.408-4.468) | 0.002 |
| Humidity (%) | 1.127 (0.851-1.491) | 0.404 |  | .. | .. |
| Wind speed (mph) | 0.962 (0.777-1.190) | 0.721 |  | .. | .. |
| Pressure (hPa) | 0.583 (0.346-0.982) | 0.043 |  | .. | .. |
| lag 1 |  |  |  |  |  |
| PM_10_ (μg/m^3^) | 0.928 (0.708-1.216) | 0.588 |  | .. | .. |
| SO_2_ (μg/m^3^) | 0.941 (0.716-1.238) | 0.665 |  | .. | .. |
| CO (mg/m^3^) | 0.880 (0.724-1.071) | 0.203 |  | .. | .. |
| NO_2_ (μg/m^3^) | 0.945 (0.741-1.204) | 0.646 |  | .. | .. |
| O_3_ (μg/m^3^) | 0.987 (0.735-1.325) | 0.930 |  | .. | .. |
| Temperature (℃) | 2.924 (1.598-5.348) | <0.001 |  | 2.924 (1.598-5.348) | <0.001 |
| Humidity (%) | 1.095 (0.810-1.481) | 0.556 |  | .. | .. |
| Wind speed (mph) | 0.891 (0.718-1.107) | 0.298 |  | .. | .. |
| Pressure (hPa) | 0.567 (0.332-0.968) | 0.038 |  | .. | .. |
| lag 2 |  |  |  |  |  |
| PM_10_ (μg/m^3^) | 1.047 (0.807-1.359) | 0.727 |  | .. | .. |
| SO_2_ (μg/m^3^) | 1.054 (0.810-1.371) | 0.697 |  | .. | .. |
| CO (mg/m^3^) | 0.940 (0.776-1.139) | 0.528 |  | .. | .. |
| NO_2_ (μg/m^3^) | 0.996 (0.782-1.268) | 0.972 |  | .. | .. |
| O_3_ (μg/m^3^) | 0.964 (0.725-1.281) | 0.800 |  | .. | .. |
| Temperature (℃) | 2.677 (1.473-4.866) | 0.001 |  | 2.677 (1.473-4.866) | 0.001 |
| Humidity (%) | 1.189 (0.893-1.583) | 0.236 |  | .. | .. |
| Wind speed (mph) | 0.823 (0.657-1.030) | 0.089 |  | .. | .. |
| Pressure (hPa) | 0.552 (0.323-0.944) | 0.030 |  | .. | .. |
| lag 3 |  |  |  |  |  |
| PM_10_ (μg/m^3^) | 1.049 (0.800-1.375) | 0.728 |  | .. | .. |
| SO_2_ (μg/m^3^) | 1.048 (0.804-1.366) | 0.729 |  | .. | .. |
| CO (mg/m^3^) | 0.885 (0.725-1.080) | 0.228 |  | .. | .. |
| NO_2_ (μg/m^3^) | 0.982 (0.765-1.261) | 0.888 |  | .. | .. |
| O_3_ (μg/m^3^) | 0.990 (0.747-1.312) | 0.944 |  | .. | .. |
| Temperature (℃) | 2.040 (1.128-3.689) | 0.018 |  | 2.040 (1.128-3.689) | 0.018 |
| Humidity (%) | 1.029 (0.785-1.349) | 0.836 |  | .. | .. |
| Wind speed (mph) | 0.812 (0.648-1.016) | 0.069 |  | .. | .. |
| Pressure (hPa) | 0.686 (0.403-1.166) | 0.164 |  | .. | .. |
| lag 4 |  |  |  |  |  |
| PM_10_ (μg/m^3^) | 1.149 (0.876-1.508) | 0.315 |  | .. | .. |
| SO_2_ (μg/m^3^) | 1.218 (0.951-1.560) | 0.119 |  | .. | .. |
| CO (mg/m^3^) | 0.964 (0.795-1.169) | 0.707 |  | .. | .. |
| NO_2_ (μg/m^3^) | 1.061 (0.819-1.375) | 0.654 |  | .. | .. |
| O_3_ (μg/m^3^) | 1.218 (0.918-1.616) | 0.171 |  | .. | .. |
| Temperature (℃) | 2.103 (1.142-3.874) | 0.017 |  | 2.103 (1.142-3.874) | 0.017 |
| Humidity (%) | 0.907 (0.691-1.190) | 0.482 |  | .. | .. |
| Wind speed (mph) | 0.877 (0.701-1.096) | 0.248 |  | .. | .. |
| Pressure (hPa) | 0.745 (0.427-1.298) | 0.298 |  | .. | .. |
| lag 5 |  |  |  |  |  |
| PM_10_ (μg/m^3^) | 1.454 (1.112-1.901) | 0.006 |  | .. | .. |
| SO_2_ (μg/m^3^) | 1.605 (1.249-2.064) | <0.001 |  | 1.605 (1.249-2.064) | <0.001 |
| CO (mg/m^3^) | 1.017 (0.843-1.227) | 0.861 |  | .. | .. |
| NO_2_ (μg/m^3^) | 1.364 (1.075-1.732) | 0.011 |  | .. | .. |
| O_3_ (μg/m^3^) | 1.389 (1.047-1.842) | 0.023 |  | .. | .. |
| Temperature (℃) | 2.211 (1.196-4.088) | 0.011 |  | .. | .. |
| Humidity (%) | 0.835 (0.638-1.092) | 0.187 |  | .. | .. |
| Wind speed (mph) | 0.819 (0.654-1.024) | 0.080 |  | .. | .. |
| Pressure (hPa) | 0.783 (0.445-1.376) | 0.395 |  | .. | .. |
| lag 6 |  |  |  |  |  |
| PM_10_ (μg/m^3^) | 1.354 (1.042-1.760) | 0.023 |  | .. | .. |
| SO_2_ (μg/m^3^) | 1.495 (1.151-1.943) | 0.003 |  | 1.495 (1.151-1.943) | 0.003 |
| CO (mg/m^3^) | 1.004 (0.836-1.207) | 0.963 |  | .. | .. |
| NO_2_ (μg/m^3^) | 1.302 (1.033-1.640) | 0.025 |  | .. | .. |
| O_3_ (μg/m^3^) | 1.295 (0.974-1.722) | 0.075 |  | .. | .. |
| Temperature (℃) | 1.942 (1.062-3.550) | 0.031 |  | .. | .. |
| Humidity (%) | 0.823 (0.624-1.086) | 0.169 |  | .. | .. |
| Wind speed (mph) | 0.829 (0.661-1.039) | 0.104 |  | .. | .. |
| Pressure (hPa) | 0.846 (0.488-1.466) | 0.551 |  | .. | .. |
| lag 7 |  |  |  |  |  |
| PM_10_ (μg/m^3^) | 1.196 (0.93-1.539) | 0.163 |  | .. | .. |
| SO_2_ (μg/m^3^) | 1.342 (1.043-1.726) | 0.022 |  | 1.342 (1.043-1.726) | 0.022 |
| CO (mg/m^3^) | 1.013 (0.843-1.217) | 0.888 |  | .. | .. |
| NO_2_ (μg/m^3^) | 1.198 (0.947-1.515) | 0.132 |  | .. | .. |
| O_3_ (μg/m^3^) | 1.070 (0.804-1.424) | 0.642 |  | .. | .. |
| Temperature (℃) | 1.653 (0.920-2.972) | 0.093 |  | .. | .. |
| Humidity (%) | 0.926 (0.698-1.228) | 0.592 |  | .. | .. |
| Wind speed (mph) | 0.901 (0.728-1.115) | 0.337 |  | .. | .. |
| Pressure (hPa) | 0.808 (0.469-1.392) | 0.443 |  | .. | .. |

Abbreviations: IQR, interquartile range; PM_10_, coarse particulate matter; OR, odds ratio; CI, confidence interval; mph, mile per hour; hPa, hectopascal.
