# Supplementary material for: Environmental Risk Factors for Talaromycosis Hospitalizations of HIV-Infected Patients in Guangzhou, China: Case Crossover Study
Source: Front Med (Lausanne). 2021 Nov 22;8:731188. doi: 10.3389/fmed.2021.731188 (PMC8645774; doi:10.3389/fmed.2021.731188)
Supplement: Supplementary Table S5 — Associations between talaromycosis hospitalizations of HIV-infected patients with pulmonary tuberculosis and an IQR increase in environmental variables. [file Table_5.DOCX]

Table S5. Associations between talaromycosis hospitalizations of HIV-infected patients with pulmonary tuberculosis and an IQR increase in environmental variables.

| Variable | Univariate analysis | |  | Multivariate analysis | |
| --- | --- | --- | --- | --- | --- |
|  | OR (95% CI) | P value |  | OR (95% CI) | P value |
| lag 0 |  |  |  |  |  |
| PM_10_ (μg/m^3^) | 0.818 (0.595-1.125) | 0.217 |  | .. | .. |
| SO_2_ (μg/m^3^) | 0.871 (0.640-1.185) | 0.378 |  | .. | .. |
| CO (mg/m^3^) | 0.989 (0.791-1.237) | 0.925 |  | .. | .. |
| NO_2_ (μg/m^3^) | 0.859 (0.641-1.153) | 0.312 |  | .. | .. |
| O_3_ (μg/m^3^) | 1.089 (0.795-1.493) | 0.595 |  | .. | .. |
| Temperature (℃) | 2.228 (1.157-4.292) | 0.017 |  | .. | .. |
| Humidity (%) | 1.129 (0.819-1.558) | 0.459 |  | .. | .. |
| Wind speed (mph) | 0.942 (0.738-1.201) | 0.627 |  | .. | .. |
| Pressure (hPa) | 0.439 (0.248-0.779) | 0.005 |  | 0.439 (0.248-0.779) | 0.005 |
| lag 1 |  |  |  |  |  |
| PM_10_ (μg/m^3^) | 0.870 (0.638-1.187) | 0.381 |  | .. | .. |
| SO_2_ (μg/m^3^) | 0.897 (0.664-1.212) | 0.479 |  | .. | .. |
| CO (mg/m^3^) | 1.002 (0.813-1.234) | 0.986 |  | .. | .. |
| NO_2_ (μg/m^3^) | 0.861 (0.645-1.150) | 0.311 |  | .. | .. |
| O_3_ (μg/m^3^) | 1.103 (0.811-1.500) | 0.533 |  | .. | .. |
| Temperature (℃) | 2.284 (1.168-4.466) | 0.016 |  | .. | .. |
| Humidity (%) | 1.020 (0.726-1.435) | 0.907 |  | .. | .. |
| Wind speed (mph) | 1.001 (0.783-1.279) | 0.996 |  | .. | .. |
| Pressure (hPa) | 0.462 (0.260-0.821) | 0.008 |  | 0.462 (0.260-0.821) | 0.008 |
| lag 2 |  |  |  |  |  |
| PM_10_ (μg/m^3^) | 0.818 (0.599-1.118) | 0.208 |  | .. | .. |
| SO_2_ (μg/m^3^) | 0.938 (0.688-1.279) | 0.687 |  | .. | .. |
| CO (mg/m^3^) | 1.006 (0.811-1.248) | 0.956 |  | .. | .. |
| NO_2_ (μg/m^3^) | 0.920 (0.692-1.224) | 0.568 |  | .. | .. |
| O_3_ (μg/m^3^) | 1.069 (0.787-1.452) | 0.670 |  | .. | .. |
| Temperature (℃) | 2.227 (1.151-4.307) | 0.017 |  | 2.227 (1.151-4.307) | 0.017 |
| Humidity (%) | 1.105 (0.791-1.544) | 0.559 |  | .. | .. |
| Wind speed (mph) | 0.893 (0.699-1.141) | 0.365 |  | .. | .. |
| Pressure (hPa) | 0.511 (0.285-0.918) | 0.025 |  | .. | .. |
| lag 3 |  |  |  |  |  |
| PM_10_ (μg/m^3^) | 0.822 (0.597-1.131) | 0.229 |  | .. | .. |
| SO_2_ (μg/m^3^) | 1.003 (0.743-1.353) | 0.986 |  | .. | .. |
| CO (mg/m^3^) | 0.867 (0.687-1.094) | 0.230 |  | .. | .. |
| NO_2_ (μg/m^3^) | 0.832 (0.618-1.119) | 0.223 |  | .. | .. |
| O_3_ (μg/m^3^) | 1.067 (0.772-1.474) | 0.694 |  | .. | .. |
| Temperature (℃) | 2.339 (1.174-4.659) | 0.016 |  | 2.339 (1.174-4.659) | 0.016 |
| Humidity (%) | 1.008 (0.719-1.413) | 0.964 |  | .. | .. |
| Wind speed (mph) | 1.006 (0.801-1.262) | 0.962 |  | .. | .. |
| Pressure (hPa) | 0.542 (0.299-0.983) | 0.044 |  | .. | .. |
| lag 4 |  |  |  |  |  |
| PM_10_ (μg/m^3^) | 0.866 (0.637-1.176) | 0.356 |  | .. | .. |
| SO_2_ (μg/m^3^) | 1.164 (0.878-1.543) | 0.292 |  | .. | .. |
| CO (mg/m^3^) | 0.969 (0.775-1.210) | 0.779 |  | .. | .. |
| NO_2_ (μg/m^3^) | 0.963 (0.723-1.281) | 0.793 |  | .. | .. |
| O_3_ (μg/m^3^) | 1.138 (0.833-1.554) | 0.416 |  | .. | .. |
| Temperature (℃) | 2.180 (1.045-4.546) | 0.038 |  | 2.180 (1.045-4.546) | 0.038 |
| Humidity (%) | 1.076 (0.769-1.506) | 0.668 |  | .. | .. |
| Wind speed (mph) | 0.989 (0.774-1.264) | 0.929 |  | .. | .. |
| Pressure (hPa) | 0.549 (0.298-1.014) | 0.055 |  | .. | .. |
| lag 5 |  |  |  |  |  |
| PM_10_ (μg/m^3^) | 0.909 (0.672-1.228) | 0.534 |  | .. | .. |
| SO_2_ (μg/m^3^) | 1.108 (0.829-1.480) | 0.490 |  | .. | .. |
| CO (mg/m^3^) | 0.956 (0.766-1.195) | 0.695 |  | .. | .. |
| NO_2_ (μg/m^3^) | 1.037 (0.783-1.375) | 0.799 |  | .. | .. |
| O_3_ (μg/m^3^) | 0.920 (0.661-1.280) | 0.621 |  | .. | .. |
| Temperature (℃) | 2.074 (0.991-4.337) | 0.053 |  | .. | .. |
| Humidity (%) | 1.150 (0.818-1.615) | 0.421 |  | .. | .. |
| Wind speed (mph) | 0.952 (0.739-1.227) | 0.704 |  | .. | .. |
| Pressure (hPa) | 0.534 (0.287-0.991) | 0.047 |  | 0.534 (0.287-0.991) | 0.047 |
| lag 6 |  |  |  |  |  |
| PM_10_ (μg/m^3^) | 0.943 (0.701-1.268) | 0.697 |  | .. | .. |
| SO_2_ (μg/m^3^) | 1.012 (0.757-1.353) | 0.934 |  | .. | .. |
| CO (mg/m^3^) | 1.063 (0.860-1.314) | 0.574 |  | .. | .. |
| NO_2_ (μg/m^3^) | 1.123 (0.852-1.480) | 0.410 |  | .. | .. |
| O_3_ (μg/m^3^) | 0.849 (0.601-1.198) | 0.350 |  | .. | .. |
| Temperature (℃) | 1.712 (0.841-3.486) | 0.138 |  | .. | .. |
| Humidity (%) | 1.252 (0.894-1.755) | 0.191 |  | .. | .. |
| Wind speed (mph) | 0.849 (0.659-1.095) | 0.207 |  | .. | .. |
| Pressure (hPa) | 0.582 (0.317-1.066) | 0.080 |  | .. | .. |
| lag 7 |  |  |  |  |  |
| PM_10_ (μg/m^3^) | 0.911 (0.680-1.221) | 0.533 |  | .. | .. |
| SO_2_ (μg/m^3^) | 1.036 (0.771-1.393) | 0.814 |  | .. | .. |
| CO (mg/m^3^) | 0.994 (0.797-1.239) | 0.955 |  | .. | .. |
| NO_2_ (μg/m^3^) | 1.040 (0.796-1.360) | 0.772 |  | .. | .. |
| O_3_ (μg/m^3^) | 1.038 (0.754-1.429) | 0.818 |  | .. | .. |
| Temperature (℃) | 1.577 (0.794-3.131) | 0.193 |  | .. | .. |
| Humidity (%) | 1.037 (0.750-1.433) | 0.826 |  | .. | .. |
| Wind speed (mph) | 0.834 (0.641-1.085) | 0.176 |  | .. | .. |
| Pressure (hPa) | 0.843 (0.462-1.538) | 0.577 |  | .. | .. |

Abbreviations: IQR, interquartile range; PM_10_, coarse particulate matter; OR, odds ratio; CI, confidence interval; mph, mile per hour; hPa, hectopascal.
