# Supplementary material for: Environmental Risk Factors for Talaromycosis Hospitalizations of HIV-Infected Patients in Guangzhou, China: Case Crossover Study
Source: Front Med (Lausanne). 2021 Nov 22;8:731188. doi: 10.3389/fmed.2021.731188 (PMC8645774; doi:10.3389/fmed.2021.731188)
Supplement: Supplementary Table S6 — Associations between talaromycosis hospitalizations of HIV-infected patients with chronic hepatitis B or C and an IQR increase in environmental variables. [file Table_6.DOCX]

Table S6. Associations between talaromycosis hospitalizations of HIV-infected patients with chronic hepatitis B or C and an IQR increase in environmental variables.

| Variable | Univariate analysis | |  | Multivariate analysis | |
| --- | --- | --- | --- | --- | --- |
|  | OR (95% CI) | P value |  | OR (95% CI) | P value |
| lag 0 |  |  |  |  |  |
| PM_10_ (μg/m^3^) | 0.937 (0.716-1.226) | 0.633 |  | .. | .. |
| SO_2_ (μg/m^3^) | 0.978 (0.732-1.308) | 0.882 |  | .. | .. |
| CO (mg/m^3^) | 0.995 (0.819-1.209) | 0.960 |  | .. | .. |
| NO_2_ (μg/m^3^) | 0.997 (0.783-1.271) | 0.984 |  | .. | .. |
| O_3_ (μg/m^3^) | 1.029 (0.752-1.408) | 0.857 |  | .. | .. |
| Temperature (℃) | 2.526 (1.320-4.837) | 0.005 |  | 2.526 (1.320-4.837) | 0.005 |
| Humidity (%) | 1.217 (0.908-1.631) | 0.189 |  | .. | .. |
| Wind speed (mph) | 0.972 (0.775-1.218) | 0.803 |  | .. | .. |
| Pressure (hPa) | 0.788 (0.462-1.344) | 0.381 |  | .. | .. |
| lag 1 |  |  |  |  |  |
| PM_10_ (μg/m^3^) | 0.857 (0.647-1.133) | 0.279 |  | .. | .. |
| SO_2_ (μg/m^3^) | 0.897 (0.666-1.208) | 0.475 |  | .. | .. |
| CO (mg/m^3^) | 0.918 (0.755-1.116) | 0.390 |  | .. | .. |
| NO_2_ (μg/m^3^) | 0.932 (0.727-1.196) | 0.582 |  | .. | .. |
| O_3_ (μg/m^3^) | 0.891 (0.655-1.212) | 0.463 |  | .. | .. |
| Temperature (℃) | 2.666 (1.401-5.074) | 0.003 |  | 2.666 (1.401-5.074) | 0.003 |
| Humidity (%) | 1.285 (0.960-1.722) | 0.092 |  | .. | .. |
| Wind speed (mph) | 0.877 (0.689-1.115) | 0.283 |  | .. | .. |
| Pressure (hPa) | 0.674 (0.396-1.146) | 0.145 |  | .. | .. |
| lag 2 |  |  |  |  |  |
| PM_10_ (μg/m^3^) | 0.945 (0.714-1.250) | 0.690 |  | .. | .. |
| SO_2_ (μg/m^3^) | 1.086 (0.811-1.453) | 0.581 |  | .. | .. |
| CO (mg/m^3^) | 0.992 (0.820-1.200) | 0.936 |  | .. | .. |
| NO_2_ (μg/m^3^) | 0.884 (0.682-1.147) | 0.355 |  | .. | .. |
| O_3_ (μg/m^3^) | 0.836 (0.615-1.136) | 0.252 |  | .. | .. |
| Temperature (℃) | 2.581 (1.341-4.966) | 0.005 |  | 2.581 (1.341-4.966) | 0.005 |
| Humidity (%) | 1.256 (0.945-1.669) | 0.116 |  | .. | .. |
| Wind speed (mph) | 0.890 (0.699-1.132) | 0.341 |  | .. | .. |
| Pressure (hPa) | 0.540 (0.318-0.917) | 0.023 |  | .. | .. |
| lag 3 |  |  |  |  |  |
| PM_10_ (μg/m^3^) | 1.042 (0.794-1.367) | 0.769 |  | .. | .. |
| SO_2_ (μg/m^3^) | 1.098 (0.814-1.481) | 0.539 |  | .. | .. |
| CO (mg/m^3^) | 0.971 (0.803-1.174) | 0.760 |  | .. | .. |
| NO_2_ (μg/m^3^) | 0.900 (0.697-1.162) | 0.419 |  | .. | .. |
| O_3_ (μg/m^3^) | 0.905 (0.665-1.232) | 0.526 |  | .. | .. |
| Temperature (℃) | 2.117 (1.132-3.958) | 0.019 |  | .. | .. |
| Humidity (%) | 1.190 (0.895-1.580) | 0.231 |  | .. | .. |
| Wind speed (mph) | 0.980 (0.787-1.221) | 0.860 |  | .. | .. |
| Pressure (hPa) | 0.519 (0.305-0.882) | 0.015 |  | 0.519 (0.305-0.882) | 0.015 |
| lag 4 |  |  |  |  |  |
| PM_10_ (μg/m^3^) | 1.003 (0.762-1.320) | 0.983 |  | .. | .. |
| SO_2_ (μg/m^3^) | 1.194 (0.908-1.571) | 0.204 |  | .. | .. |
| CO (mg/m^3^) | 0.979 (0.808-1.187) | 0.833 |  | .. | .. |
| NO_2_ (μg/m^3^) | 0.932 (0.727-1.194) | 0.577 |  | .. | .. |
| O_3_ (μg/m^3^) | 1.105 (0.825-1.479) | 0.502 |  | .. | .. |
| Temperature (℃) | 1.973 (1.051-3.704) | 0.034 |  | 1.973 (1.051-3.704) | 0.034 |
| Humidity (%) | 0.962 (0.732-1.263) | 0.778 |  | .. | .. |
| Wind speed (mph) | 0.930 (0.744-1.162) | 0.521 |  | .. | .. |
| Pressure (hPa) | 0.669 (0.391-1.146) | 0.144 |  | .. | .. |
| lag 5 |  |  |  |  |  |
| PM_10_ (μg/m^3^) | 0.930 (0.711-1.217) | 0.599 |  | .. | .. |
| SO_2_ (μg/m^3^) | 1.166 (0.881-1.543) | 0.282 |  | .. | .. |
| CO (mg/m^3^) | 0.910 (0.749-1.106) | 0.343 |  | .. | .. |
| NO_2_ (μg/m^3^) | 0.928 (0.726-1.187) | 0.553 |  | .. | .. |
| O_3_ (μg/m^3^) | 1.069 (0.806-1.417) | 0.643 |  | .. | .. |
| Temperature (℃) | 1.767 (0.941-3.319) | 0.077 |  | .. | .. |
| Humidity (%) | 0.968 (0.741-1.265) | 0.813 |  | .. | .. |
| Wind speed (mph) | 0.987 (0.795-1.227) | 0.909 |  | .. | .. |
| Pressure (hPa) | 0.901 (0.530-1.530) | 0.698 |  | .. | .. |
| lag 6 |  |  |  |  |  |
| PM_10_ (μg/m^3^) | 0.869 (0.657-1.149) | 0.324 |  | .. | .. |
| SO_2_ (μg/m^3^) | 1.078 (0.804-1.445) | 0.615 |  | .. | .. |
| CO (mg/m^3^) | 0.891 (0.732-1.085) | 0.250 |  | .. | .. |
| NO_2_ (μg/m^3^) | 0.945 (0.738-1.211) | 0.656 |  | .. | .. |
| O_3_ (μg/m^3^) | 0.971 (0.732-1.288) | 0.838 |  | .. | .. |
| Temperature (℃) | 1.537 (0.829-2.850) | 0.173 |  | .. | .. |
| Humidity (%) | 0.997 (0.755-1.316) | 0.983 |  | .. | .. |
| Wind speed (mph) | 0.965 (0.772-1.207) | 0.755 |  | .. | .. |
| Pressure (hPa) | 0.978 (0.579-1.651) | 0.933 |  | .. | .. |
| lag 7 |  |  |  |  |  |
| PM_10_ (μg/m^3^) | 0.762 (0.569-1.019) | 0.067 |  | .. | .. |
| SO_2_ (μg/m^3^) | 0.948 (0.703-1.277) | 0.724 |  | .. | .. |
| CO (mg/m^3^) | 0.913 (0.747-1.116) | 0.374 |  | .. | .. |
| NO_2_ (μg/m^3^) | 0.823 (0.631-1.075) | 0.153 |  | .. | .. |
| O_3_ (μg/m^3^) | 0.828 (0.605-1.134) | 0.240 |  | .. | .. |
| Temperature (℃) | 1.197 (0.663-2.161) | 0.551 |  | .. | .. |
| Humidity (%) | 1.033 (0.776-1.376) | 0.823 |  | .. | .. |
| Wind speed (mph) | 1.052 (0.843-1.312) | 0.653 |  | .. | .. |
| Pressure (hPa) | 1.038 (0.613-1.758) | 0.889 |  | .. | .. |

Abbreviations: IQR, interquartile range; PM_10_, coarse particulate matter; OR, odds ratio; CI, confidence interval; mph, mile per hour; hPa, hectopascal.
