# Supplementary material for: Environmental Risk Factors for Talaromycosis Hospitalizations of HIV-Infected Patients in Guangzhou, China: Case Crossover Study
Source: Front Med (Lausanne). 2021 Nov 22;8:731188. doi: 10.3389/fmed.2021.731188 (PMC8645774; doi:10.3389/fmed.2021.731188)
Supplement: Supplementary Table S7 — Demographic and clinical features of patients stratified by age. [file Table_7.DOCX]

Table S7. Demographic and clinical features of patients stratified by age.

| Parameter |  | < 50 yrs (n=729) | ≥50 yrs (n=190) | P value |
| --- | --- | --- | --- | --- |
| Male | | 592 (81.2%) | 159 (83.7%) | 0.431 |
| CD4 cell count, cells/μL | | 9 (4-20) | 8 (4-19) | 0.066 |
| Co-infection | C. neoformans infection | 23 (3.2%) | 6 (3.2%) | 0.998 |
|  | Candidiasis | 295 (40.5%) | 91 (47.9%) | 0.065 |
|  | Pneumocystis pneumonia | 143 (19.6%) | 40 (21.1%) | 0.659 |
|  | Pulmonary tuberculosis | 109 (15.0%) | 32 (16.8%) | 0.520 |
|  | Chronic hepatitis B or C | 131 (18.0%) | 27 (14.2%) | 0.221 |
| Comorbidity | Diabetes | 11 (1.5%) | 10 (5.3%) | **0.005** |
|  | Liver cirrhosis | 14 (1.9%) | 8 (4.2%) | 0.116 |

Data are in absolute count (%) for categorical variables and median (interquartile range [IQR]) for continuous data. Student's t-tests were applied to continuous variables, and χ^2^ tests were used for categorical variables. Statistically significant differences (P<0.05) are shown in boldface.
