# Supplementary material for: Environmental Risk Factors for Talaromycosis Hospitalizations of HIV-Infected Patients in Guangzhou, China: Case Crossover Study
Source: Front Med (Lausanne). 2021 Nov 22;8:731188. doi: 10.3389/fmed.2021.731188 (PMC8645774; doi:10.3389/fmed.2021.731188)
Supplement: Supplementary Table S8 — Associations between talaromycosis hospitalizations of younger patients and an IQR increase in environmental variables. [file Table_8.DOCX]

Table S8. Associations between talaromycosis hospitalizations of younger patients and an IQR increase in environmental variables.

| Variable | Univariate analysis | |  | Multivariate analysis | |
| --- | --- | --- | --- | --- | --- |
|  | OR (95% CI) | P value |  | OR (95% CI) | P value |
| lag 0 |  |  |  |  |  |
| PM_10_ (μg/m^3^) | 0.954 (0.835-1.090) | 0.490 |  | .. | .. |
| SO_2_ (μg/m^3^) | 0.933 (0.812-1.071) | 0.323 |  | .. | .. |
| CO (mg/m^3^) | 0.953 (0.864-1.052) | 0.341 |  | .. | .. |
| NO_2_ (μg/m^3^) | 0.980 (0.867-1.106) | 0.740 |  | .. | .. |
| O_3_ (μg/m^3^) | 0.954 (0.828-1.099) | 0.516 |  | .. | .. |
| Temperature (℃) | 2.092 (1.560-2.805) | <0.001 |  | 2.092 (1.560-2.805) | <0.001 |
| Humidity (%) | 1.140 (0.997-1.305) | 0.056 |  | .. | .. |
| Wind speed (mph) | 0.973 (0.877-1.079) | 0.599 |  | .. | .. |
| Pressure (hPa) | 0.715 (0.555-0.922) | 0.010 |  | .. | .. |
| lag 1 |  |  |  |  |  |
| PM_10_ (μg/m^3^) | 1.000 (0.876-1.142) | 1.000 |  | .. | .. |
| SO_2_ (μg/m^3^) | 0.990 (0.864-1.134) | 0.881 |  | .. | .. |
| CO (mg/m^3^) | 0.987 (0.896-1.087) | 0.788 |  | .. | .. |
| NO_2_ (μg/m^3^) | 1.005 (0.891-1.134) | 0.932 |  | .. | .. |
| O_3_ (μg/m^3^) | 0.968 (0.841-1.114) | 0.650 |  | .. | .. |
| Temperature (℃) | 2.182 (1.616-2.947) | <0.001 |  | 2.182 (1.616-2.947) | <0.001 |
| Humidity (%) | 1.138 (0.992-1.305) | 0.064 |  | .. | .. |
| Wind speed (mph) | 0.932 (0.836-1.038) | 0.200 |  | .. | .. |
| Pressure (hPa) | 0.703 (0.544-0.909) | 0.007 |  | .. | .. |
| lag 2 |  |  |  |  |  |
| PM_10_ (μg/m^3^) | 1.104 (0.97-1.257) | 0.133 |  | .. | .. |
| SO_2_ (μg/m^3^) | 1.115 (0.972-1.28) | 0.120 |  | .. | .. |
| CO (mg/m^3^) | 1.035 (0.942-0.138) | 0.473 |  | .. | .. |
| NO_2_ (μg/m^3^) | 1.078 (0.959-0.213) | 0.208 |  | .. | .. |
| O_3_ (μg/m^3^) | 1.022 (0.890-0.173) | 0.762 |  | .. | .. |
| Temperature (℃) | 2.214 (1.636-2.994) | <0.001 |  | 2.214 (1.636-2.994) | <0.001 |
| Humidity (%) | 1.059 (0.925-0.211) | 0.406 |  | .. | .. |
| Wind speed (mph) | 0.879 (0.788-0.980) | 0.020 |  | .. | .. |
| Pressure (hPa) | 0.678 (0.525-0.877) | 0.003 |  | .. | .. |
| lag 3 |  |  |  |  |  |
| PM_10_ (μg/m^3^) | 1.025 (0.895-1.174) | 0.718 |  | .. | .. |
| SO_2_ (μg/m^3^) | 1.018 (0.886-1.171) | 0.797 |  | .. | .. |
| CO (mg/m^3^) | 0.949 (0.86-1.047) | 0.293 |  | .. | .. |
| NO_2_ (μg/m^3^) | 0.992 (0.876-1.124) | 0.905 |  | .. | .. |
| O_3_ (μg/m^3^) | 1.037 (0.900-1.194) | 0.619 |  | .. | .. |
| Temperature (℃) | 1.876 (1.392-2.528) | <0.001 |  | 1.876 (1.392-2.528) | <0.001 |
| Humidity (%) | 0.955 (0.835-1.092) | 0.499 |  | .. | .. |
| Wind speed (mph) | 0.887 (0.796-0.988) | 0.029 |  | .. | .. |
| Pressure (hPa) | 0.722 (0.558-0.935) | 0.013 |  | .. | .. |
| lag 4 |  |  |  |  |  |
| PM_10_ (μg/m^3^) | 0.991 (0.865-1.135) | 0.892 |  | .. | .. |
| SO_2_ (μg/m^3^) | 0.997 (0.869-1.144) | 0.966 |  | .. | .. |
| CO (mg/m^3^) | 0.923 (0.837-1.019) | 0.113 |  | .. | .. |
| NO_2_ (μg/m^3^) | 0.939 (0.827-1.066) | 0.333 |  | .. | .. |
| O_3_ (μg/m^3^) | 1.025 (0.891-1.180) | 0.730 |  | .. | .. |
| Temperature (℃) | 1.672 (1.244-2.248) | 0.001 |  | 1.672 (1.244-2.248) | 0.001 |
| Humidity (%) | 0.919 (0.805-1.050) | 0.213 |  | .. | .. |
| Wind speed (mph) | 0.959 (0.861-1.067) | 0.441 |  | .. | .. |
| Pressure (hPa) | 0.786 (0.607-1.019) | 0.069 |  | .. | .. |
| lag 5 |  |  |  |  |  |
| PM_10_ (μg/m^3^) | 0.964 (0.843-1.102) | 0.589 |  | .. | .. |
| SO_2_ (μg/m^3^) | 1.033 (0.903-1.181) | 0.633 |  | .. | .. |
| CO (mg/m^3^) | 0.924 (0.838-1.019) | 0.114 |  | .. | .. |
| NO_2_ (μg/m^3^) | 0.942 (0.832-1.066) | 0.344 |  | .. | .. |
| O_3_ (μg/m^3^) | 0.996 (0.866-1.145) | 0.951 |  | .. | .. |
| Temperature (℃) | 1.609 (1.198-2.160) | 0.002 |  | 1.609 (1.198-2.160) | 0.002 |
| Humidity (%) | 0.928 (0.814-1.057) | 0.261 |  | .. | .. |
| Wind speed (mph) | 1.021 (0.920-1.134) | 0.692 |  | .. | .. |
| Pressure (hPa) | 0.833 (0.643-1.079) | 0.166 |  | .. | .. |
| lag 6 |  |  |  |  |  |
| PM_10_ (μg/m^3^) | 0.955 (0.836-1.090) | 0.495 |  | .. | .. |
| SO_2_ (μg/m^3^) | 0.938 (0.818-1.075) | 0.358 |  | .. | .. |
| CO (mg/m^3^) | 0.966 (0.878-1.063) | 0.481 |  | .. | .. |
| NO_2_ (μg/m^3^) | 0.942 (0.834-1.063) | 0.333 |  | .. | .. |
| O_3_ (μg/m^3^) | 0.930 (0.807-1.072) | 0.316 |  | .. | .. |
| Temperature (℃) | 1.606 (1.194-2.161) | 0.002 |  | 1.606 (1.194-2.161) | 0.002 |
| Humidity (%) | 1.011 (0.886-1.154) | 0.873 |  | .. | .. |
| Wind speed (mph) | 1.027 (0.925-1.139) | 0.623 |  | .. | .. |
| Pressure (hPa) | 0.806 (0.623-1.044) | 0.102 |  | .. | .. |
| lag 7 |  |  |  |  |  |
| PM_10_ (μg/m^3^) | 0.966 (0.847-1.100) | 0.599 |  | .. | .. |
| SO_2_ (μg/m^3^) | 0.946 (0.824-1.086) | 0.431 |  | .. | .. |
| CO (mg/m^3^) | 1.011 (0.918-1.113) | 0.825 |  | .. | .. |
| NO_2_ (μg/m^3^) | 0.948 (0.840-1.070) | 0.386 |  | .. | .. |
| O_3_ (μg/m^3^) | 0.883 (0.765-1.020) | 0.090 |  | .. | .. |
| Temperature (℃) | 1.598 (1.190-2.146) | 0.002 |  | 1.598 (1.190-2.146) | 0.002 |
| Humidity (%) | 1.123 (0.980-1.287) | 0.096 |  | .. | .. |
| Wind speed (mph) | 0.970 (0.874-1.076) | 0.568 |  | .. | .. |
| Pressure (hPa) | 0.759 (0.584-0.986) | 0.039 |  | .. | .. |

Abbreviations: IQR, interquartile range; PM_10_, coarse particulate matter; OR, odds ratio; CI, confidence interval; mph, mile per hour; hPa, hectopascal.
