# Supplementary material for: Environmental Risk Factors for Talaromycosis Hospitalizations of HIV-Infected Patients in Guangzhou, China: Case Crossover Study
Source: Front Med (Lausanne). 2021 Nov 22;8:731188. doi: 10.3389/fmed.2021.731188 (PMC8645774; doi:10.3389/fmed.2021.731188)
Supplement: Supplementary Table S9 — Associations between talaromycosis hospitalizations of older patients and an IQR increase in environmental variables. [file Table_9.DOCX]

Table S9. Associations between talaromycosis hospitalizations of older patients and an IQR increase in environmental variables.

| Variable | Univariate analysis | |  | Multivariate analysis | |
| --- | --- | --- | --- | --- | --- |
|  | OR (95% CI) | P value |  | OR (95% CI) | P value |
| lag 0 |  |  |  |  |  |
| PM_10_ (μg/m^3^) | 1.016 (0.771-1.339) | 0.911 |  | .. | .. |
| SO_2_ (μg/m^3^) | 1.052 (0.789-1.402) | 0.731 |  | .. | .. |
| CO (mg/m^3^) | 1.187 (0.977-1.442) | 0.084 |  | .. | .. |
| NO_2_ (μg/m^3^) | 1.063 (0.828-1.364) | 0.632 |  | .. | .. |
| O_3_ (μg/m^3^) | 0.952 (0.725-1.250) | 0.721 |  | .. | .. |
| Temperature (℃) | 2.110 (1.213-3.670) | 0.008 |  | .. | .. |
| Humidity (%) | 1.231 (0.945-1.603) | 0.123 |  | .. | .. |
| Wind speed (mph) | 0.936 (0.755-1.160) | 0.547 |  | .. | .. |
| Pressure (hPa) | 0.468 (0.281-0.778) | 0.003 |  | 0.468 (0.281-0.778) | 0.003 |
| lag 1 |  |  |  |  |  |
| PM_10_ (μg/m^3^) | 0.951 (0.717-1.262) | 0.729 |  | .. | .. |
| SO_2_ (μg/m^3^) | 1.005 (0.751-1.345) | 0.974 |  | .. | .. |
| CO (mg/m^3^) | 1.074 (0.875-1.318) | 0.496 |  | .. | .. |
| NO_2_ (μg/m^3^) | 0.992 (0.769-1.280) | 0.951 |  | .. | .. |
| O_3_ (μg/m^3^) | 0.998 (0.759-1.311) | 0.986 |  | .. | .. |
| Temperature (℃) | 1.932 (1.108-3.371) | 0.020 |  | .. | .. |
| Humidity (%) | 1.270 (0.971-1.662) | 0.081 |  | .. | .. |
| Wind speed (mph) | 0.912 (0.736-1.130) | 0.401 |  | .. | .. |
| Pressure (hPa) | 0.451 (0.269-0.755) | 0.002 |  | 0.451 (0.269-0.755) | 0.002 |
| lag 2 |  |  |  |  |  |
| PM_10_ (μg/m^3^) | 0.949 (0.714-1.261) | 0.719 |  | .. | .. |
| SO_2_ (μg/m^3^) | 1.027 (0.770-1.369) | 0.858 |  | .. | .. |
| CO (mg/m^3^) | 1.052 (0.861-1.285) | 0.620 |  | .. | .. |
| NO_2_ (μg/m^3^) | 0.979 (0.755-1.269) | 0.871 |  | .. | .. |
| O_3_ (μg/m^3^) | 0.940 (0.723-1.222) | 0.646 |  | .. | .. |
| Temperature (℃) | 1.811 (1.037-3.161) | 0.037 |  | .. | .. |
| Humidity (%) | 1.341 (1.033-1.740) | 0.027 |  | .. | .. |
| Wind speed (mph) | 0.802 (0.639-1.006) | 0.056 |  | .. | .. |
| Pressure (hPa) | 0.439 (0.259-0.745) | 0.002 |  | 0.439 (0.259-0.745) | 0.002 |
| lag 3 |  |  |  |  |  |
| PM_10_ (μg/m^3^) | 1.013 (0.766-1.340) | 0.926 |  | .. | .. |
| SO_2_ (μg/m^3^) | 1.068 (0.804-1.419) | 0.651 |  | .. | .. |
| CO (mg/m^3^) | 1.045 (0.855-1.276) | 0.670 |  | .. | .. |
| NO_2_ (μg/m^3^) | 0.924 (0.716-1.191) | 0.542 |  | .. | .. |
| O_3_ (μg/m^3^) | 1.021 (0.780-1.337) | 0.879 |  | .. | .. |
| Temperature (℃) | 1.964 (1.118-3.451) | 0.019 |  | .. | .. |
| Humidity (%) | 1.163 (0.899-1.506) | 0.250 |  | .. | .. |
| Wind speed (mph) | 0.905 (0.726-1.128) | 0.374 |  | .. | .. |
| Pressure (hPa) | 0.515 (0.304-0.872) | 0.013 |  | 0.515 (0.304-0.872) | 0.013 |
| lag 4 |  |  |  |  |  |
| PM_10_ (μg/m^3^) | 1.076 (0.813-1.424) | 0.608 |  | .. | .. |
| SO_2_ (μg/m^3^) | 1.311 (1.000-1.719) | 0.050 |  | .. | .. |
| CO (mg/m^3^) | 1.141 (0.937-1.388) | 0.189 |  | .. | .. |
| NO_2_ (μg/m^3^) | 0.946 (0.731-1.225) | 0.676 |  | .. | .. |
| O_3_ (μg/m^3^) | 1.125 (0.862-1.467) | 0.385 |  | .. | .. |
| Temperature (℃) | 2.078 (1.163-3.714) | 0.014 |  | .. | .. |
| Humidity (%) | 1.163 (0.894-1.515) | 0.261 |  | .. | .. |
| Wind speed (mph) | 0.977 (0.789-1.210) | 0.832 |  | .. | .. |
| Pressure (hPa) | 0.484 (0.285-0.821) | 0.007 |  | 0.484 (0.285-0.821) | 0.007 |
| lag 5 |  |  |  |  |  |
| PM_10_ (μg/m^3^) | 1.367 (1.034-1.807) | 0.028 |  | .. | .. |
| SO_2_ (μg/m^3^) | 1.501 (1.132-1.990) | 0.005 |  | .. | .. |
| CO (mg/m^3^) | 1.207 (0.995-1.463) | 0.056 |  | .. | .. |
| NO_2_ (μg/m^3^) | 1.110 (0.862-1.429) | 0.418 |  | .. | .. |
| O_3_ (μg/m^3^) | 1.159 (0.882-1.522) | 0.291 |  | .. | .. |
| Temperature (℃) | 3.251 (1.746-6.053) | <0.001 |  | 3.251 (1.746-6.053) | <0.001 |
| Humidity (%) | 1.037 (0.791-1.360) | 0.794 |  | .. | .. |
| Wind speed (mph) | 0.969 (0.782-1.199) | 0.769 |  | .. | .. |
| Pressure (hPa) | 0.442 (0.260-0.753) | 0.003 |  | .. | .. |
| lag 6 |  |  |  |  |  |
| PM_10_ (μg/m^3^) | 1.292 (0.978-1.707) | 0.071 |  | .. | .. |
| SO_2_ (μg/m^3^) | 1.498 (1.119-2.006) | 0.007 |  | .. | .. |
| CO (mg/m^3^) | 1.087 (0.898-1.314) | 0.391 |  | .. | .. |
| NO_2_ (μg/m^3^) | 1.197 (0.940-1.524) | 0.145 |  | .. | .. |
| O_3_ (μg/m^3^) | 1.170 (0.892-1.534) | 0.257 |  | .. | .. |
| Temperature (℃) | 3.419 (1.885-6.201) | <0.001 |  | 3.419 (1.885-6.201) | <0.001 |
| Humidity (%) | 0.962 (0.741-1.248) | 0.768 |  | .. | .. |
| Wind speed (mph) | 0.866 (0.692-1.084) | 0.209 |  | .. | .. |
| Pressure (hPa) | 0.506 (0.299-0.854) | 0.011 |  | .. | .. |
| lag 7 |  |  |  |  |  |
| PM_10_ (μg/m^3^) | 1.093 (0.830-1.440) | 0.525 |  | .. | .. |
| SO_2_ (μg/m^3^) | 1.209 (0.904-1.616) | 0.201 |  | .. | .. |
| CO (mg/m^3^) | 1.088 (0.895-1.323) | 0.398 |  | .. | .. |
| NO_2_ (μg/m^3^) | 1.140 (0.894-1.453) | 0.290 |  | .. | .. |
| O_3_ (μg/m^3^) | 1.136 (0.871-1.482) | 0.346 |  | .. | .. |
| Temperature (℃) | 2.441 (1.364-4.368) | 0.003 |  | 2.441 (1.364-4.368) | 0.003 |
| Humidity (%) | 1.042 (0.804-1.351) | 0.756 |  | .. | .. |
| Wind speed (mph) | 0.793 (0.630-1.000) | 0.050 |  | .. | .. |
| Pressure (hPa) | 0.726 (0.431-1.222) | 0.228 |  | .. | .. |

Abbreviations: IQR, interquartile range; PM_10_, coarse particulate matter; OR, odds ratio; CI, confidence interval; mph, mile per hour; hPa, hectopascal.
